# Hippocampal tau distribution in primary age‐related tauopathy: The Vantaa 85+ Study

**DOI:** 10.1002/alz.70613

**Published:** 2025-08-22

**Authors:** Sara Savola, Jarno Tuimala, Ville Kivistö, Mia Kero, Anna Raunio, Darshan Kumar, Henri Puttonen, Olli Tynninen, Anders Paetau, Tuomo Polvikoski, Benjamin Englert, Liisa Myllykangas

**Affiliations:** ^1^ Department of Pathology University of Helsinki Helsinki Finland; ^2^ Department of Pathology HUS Diagnostic Center Helsinki Finland; ^3^ Aiforia Technologies Oyj Helsinki Finland; ^4^ Translational and Clinical Research Institute Newcastle University Newcastle upon Tyne UK

**Keywords:** Alzheimer's disease, amyloid beta, artificial intelligence, CA2, cognitive impairment, hippocampus, neurofibrillary degeneration, primary age‐related tauopathy, tau

## Abstract

**INTRODUCTION:**

In contrast to Alzheimer's disease (AD), in which initial neurofibrillary tangles (NFT) are mainly limited to the (trans)entorhinal region (EC) and CA1/prosubiculum, primary age‐related tauopathy (PART) has been suggested to exhibit an early predisposition to NFTs in the hippocampal CA2 subregion.

**METHODS:**

We created an artificial intelligence model that recognizes and quantifies NFTs of three different maturity levels in different hippocampal subfields. This model was applied to a population‐based Vantaa 85+ cohort, including hippocampal tau‐immunostained sections from 210 individuals aged ≥ 85 years.

**RESULTS:**

EC and CA1, but not CA2, had significantly higher NFT density in moderate AD compared to PART. CA2/CA1 and CA2/EC NFT ratios were inversely associated with amyloid beta (Aβ) deposition in PART versus AD. The maturation process was exacerbated by Aβ in AD versus PART.

**DISCUSSION:**

Compared to AD, a CA2 conspicuous pattern of hippocampal NFTs was more common in, but not exclusive to, PART.

**Highlights:**

- Neurofibrillary tangles (NFTs) of varying maturity were studied by a new kind of artificial intelligence model in the oldest‐old.
- Hippocampal NFT distribution differed between primary age‐related tauopathy (PART) and Alzheimer's disease (AD).
- PART more often showed prominent NFT pathology in CA2 compared to AD.
- In PART, the NFT maturation process stood out in CA2 compared to other subfields.
- The NFT maturation process was aggravated by amyloid beta in AD compared to PART.

## 1 BACKGROUND

Alzheimer's disease (AD) is an aging‐related progressive neurodegenerative disease with devastating effects on the affected individual's cognition and functional ability.^1^ The neuropathological hallmark of AD is the abnormal accumulation of (1) hyperphosphorylated tau protein inside neurons as so‐called neurofibrillary tangles (NFTs) and (2) extracellular amyloid beta (Aβ) peptides as amyloid plaques.^2^ Aβ deposition and neurofibrillary changes usually progress throughout the brain in typical hierarchical neuroanatomical sequences (i.e., the Thal phases^3^ and Braak stages,^4^, ^5^ respectively). In a complex interaction alongside microglial activation, Aβ deposition and neurofibrillary changes cause synaptic dysfunction and neuronal loss, eventually leading to brain atrophy.^6^

However, it is universally acknowledged that neurofibrillary pathology can exist in the aging brain without simultaneous Aβ plaques.^7^, ^8^ In fact, pretangles can first be seen in subcortical nuclei, such as locus coeruleus and in the transentorhinal cortex in the medial temporal lobe (MTL) at a young age before the appearance of Aβ depositions.^9^ In the oldest‐old, studies show that ≈ 20% lack Aβ plaques. This is in contrast to NFTs, which can be seen in almost everyone.^9^, ^10^, ^11^, ^12^, ^13^ This is a discrepancy to (1) the neuropathologic criteria for AD set by the National Institute on Aging–Alzheimer's Association (NIA‐AA) guidelines, which require both NFTs and Aβ pathology to be present for a diagnosis of AD neuropathologic change (ADNC),^14^, ^15^ and (2) the amyloid cascade hypothesis, which theorizes that AD is a secondary tauopathy in which NFT accumulation is driven by Aβ.^16^, ^17^ Therefore, it has been proposed that neurofibrillary changes may accumulate in the MTL (and brainstem) independently from Aβ,^18^, ^19^, ^20^, ^21^, ^22^ and that this NFT+/Aβ– neuropathological pattern represents a primary tauopathy, which has been called primary age‐related tauopathy (PART).^7^ In PART, without co‐existing Aβ plaques, the neurofibrillary pathology is usually limited to the MTL, rarely spreading to other neocortical regions (i.e., limited to Braak stages I–IV).^7^, ^11^ Conversely, in classical AD, neurofibrillary pathology eventually spreads widely to the neocortex (Braak stages V–VI), usually accompanied by abundant Aβ plaques.^23^

Nonetheless, critiques of PART consider it to simply represent early AD that eventually develops Aβ plaques and progresses along the AD spectrum.^24^, ^25^, ^26^ Supporting evidence of PART being a separatable entity from classical AD includes a reverse association^27^ with the greatest genetic risk factor for AD, apolipoprotein E (*APOE*) ε4 allele,^28^ a lesser degree of cognitive decline^29^, ^30^ and differences in comorbidities,^12^, ^31^ and in the neuropathological features mentioned above. Recently, it was also suggested that PART exhibits an early susceptibility to neurofibrillary pathology in the hippocampal CA2 subregion,^32^, ^33^ as opposed to classical AD in which initial neurofibrillary pathology is mainly limited to the (trans)entorhinal region and the CA1/prosubiculum.^34^, ^35^

We previously described the frequency of PART and its associations to cognitive decline and co‐pathologies in the population‐based Vantaa 85+ Study.^12^ The objective of the current study was to explore PART further and investigate possible differences in the distribution of NFTs in hippocampal subfields between PART and ADNC. We determined if we could replicate the finding of CA2 susceptibility in PART in the population‐based Vantaa 85+ Study material. Additionally, we wanted to investigate if the maturation process of NFTs from pretangles to mature tangles^36^ differed between PART and ADNC, as this could offer some further insights into possible differences or similarities between PART and AD. To our knowledge, this has not been previously studied from the perspective of PART. To achieve these objectives, we created a new kind of artificial intelligence (AI) model that recognized NFTs of three different maturity levels and quantified NFTs in different hippocampal subfields. Results were adjusted for other comorbidities. Neuropathological factors driving cognitive decline in PART were also studied.

## 2 METHODS

### 2.1 Participants

Details on the Vantaa 85+ Study have been described previously.^37^, ^38^ Briefly, the Vantaa 85+ Study is a prospective population‐based study that began in Finland in 1991. The original cohort consisted of all individuals aged ≥ 85 years who were living in the city of Vantaa on April 1, 1991 (*n* = 601). Clinical assessment and genetic sampling were performed on most participants. A total of 304 participants were autopsied, creating a neuropathological subpopulation of 252 women and 52 men, ranging in age from 85 to 105 years at death. *APOE* genotyping^39^ and cognitive assessment^40^, ^41^ has been described previously.

### 2.2 Neuropathology

Methodological details on neuropathological assessment of the following variables have been described previously: Braak stage and Thal phase,^12^ Consortium to Establish a Registry for Alzheimer's Disease (CERAD) neuritic plaque score,^38^ hippocampal sclerosis,^42^ argyrophilic grains (AGs),^12^ limbic‐predominant age‐related TDP‐43 encephalopathy neuropathologic change (LATE‐NC),^43^ Dementia with Lewy Bodies (DLB) Consortium Lewy‐related pathology (LRP) type,^44^, ^45^ cerebral amyloid angiopathy (CAA),^46^ and sclerotic index (SI) as an assessment of arteriolosclerosis.^43^ A quantitative assessment of cortical Aβ burden has also been published previously.^47^ In short, to estimate the amount of Aβ deposition in the cerebral cortex, the average percentage of cortex covered by methenamine silver–positive plaques was calculated by assessing four different cortical regions (middle frontal, superior temporal, and middle temporal gyri and inferior parietal lobule). Evaluation of aging‐related tau astrogliopathy (ARTAG) is described in the Supplementary Methods in supporting information.

**RESEARCH IN CONTEXT**

1. **Systematic review**: The existing literature was reviewed using online sources (e.g., PubMed). It remains unclear if primary age‐related tauopathy (PART) and Alzheimer's disease (AD) are separate entities. Previous studies suggesting that PART exhibits early susceptibility to neurofibrillary tangle (NFT) pathology in the hippocampal CA2 subfield, a possible distinguishing neuropathological feature from classical AD, were recently questioned.
2. **Interpretation**: Investigating hippocampal NFT distribution in PART and AD in the Vantaa 85+ Study revealed the natural spectrum of NFT and amyloid beta (Aβ) pathology and could therefore elaborate on findings from hospital‐based studies. Examining the maturation of NFTs by an artificial intelligence–based method in PART was a novel perspective.
3. **Future directions**: Further studies using unselected cohorts and wider age groups are needed to clarify differences of the CA2 conspicuous pattern of neurofibrillary degeneration in AD versus PART, including the mechanisms on how Aβ can alter hippocampal NFT distribution.

For the current study, we used immunohistologically stained (AT8) histological slides of the right hippocampus sampled at the level of the lateral geniculate body^42^ to perform quantitative assessment of NFTs. We excluded samples in which the level of the hippocampus or orientation of the section was suboptimal and samples with technical or artifact issues. Additionally, cases with hippocampal sclerosis or large infarcts in the hippocampus were excluded. One participant was excluded due to progressive supranuclear palsy. The final number of individuals included was 210. They were divided into PART and ADNC groups based on the ABC (amyloid,^3^ Braak,^4^, ^5^ CERAD^48^) scoring system by the NIA‐AA guidelines^14^, ^15^ and the current criteria for classifying PART by Crary et al., which stratifies PART into definite PART (Braak stages I–IV and absent Aβ plaques, that is, Thal phase 0 or alternatively CERAD 0) and possible PART (Braak stages I–IV and sparse Aβ plaques, that is, Thal phases 1–2 or alternatively CERAD score 1).^7^ For the current study, PART was defined as individuals with Braak stages I through IV, no neocortical neuritic plaques (CERAD score 0), and no or minimal Aβ depositions (Thal phase 0–2). We did not group definite and possible PART separately to retain statistical power for the analysis. Individuals with ADNC were defined as follows: low ADNC (Braak stage I–IV, CERAD score 1, Thal phase 1–5), moderate ADNC (Braak stage I–IV, CERAD score 2–3, Thal phase 2–5), and high ADNC (Braak stage V–VI, CERAD score 2–3, Thal phase 3–5). Eleven individuals did not fit the above grouping criteria (two individuals with CERAD score 0, Thal phase 1–2 but Braak stage V–VI, and nine with CERAD score 0, Thal phase 3, and Braak stage I–IV).

### 2.3 AI‐assisted analysis of NFTs in hippocampal subfields

The AT8‐stained histological slides of the hippocampus were digitalized at 20× magnification using a digital slide scanner (3DHistec). The digital whole slide images (WSI) were then uploaded to Aiforia cloud (Aiforia Technologies Plc.), a cloud‐based platform on which a deep‐learning AI software can be used to create AI models for image analysis. First, to create an AI model that detects NFTs, we defined three NFT classes capturing the gradual maturation process of NFTs from initial pretangles to mature tangles, with intermediary morphologies existing between.^36^ A publication by Moloney et al.^36^ was used as guidance for classification of tangles. The three classes were pretangles (“preNFTs”), intermediary NFTs (“iNFTs”), and mature NFTs (“mNFTs”). Because the final maturity level, ghost tangles, is not easily recognized by AT8,^36^ we did not attempt to capture ghost tangles separately. The classification criteria for the cellular morphology of the three maturity classes is found in the Supplementary Methods. Second, we used the Aiforia Create training tool to create, train, and validate the AI model (Aiforia Create Version 5.5, Aiforia Technologies Plc), which was done by annotating the three NFT classes on the hippocampal WSIs on 36 training and 25 separate validation images. The process of developing the AI model is described in detail in supporting information (see Supplementary Methods and Figure S1). Third, when the AI model was finished, we performed image analysis on different hippocampal subfields (entorhinal cortex [EC], CA1–4, dentate gyrus [DG], and subiculum), which were drawn manually on the hippocampal WSIs in Aiforia Cloud by segmenting them similarly to a recent study.^49^ The criteria for hippocampal subfield segmentation are described in supporting information (see Supplementary Methods and Figure S2).

The final data produced by the AI model consisted of counts of preNFTs, iNFTs, and mNFTs from each segmented hippocampal subfield, which were then divided with the area of each individual region, yielding density scores of NFTs for the hippocampal subfields (preNFT/mm^2^, iNFT/mm^2^, mNFT/mm^2^, and these three combined as total NFT/mm^2^).

### 2.4 Statistical analysis

Statistical analyses were conducted using SPSS version 28.^50^ A Fisher exact test was used to test for associations between study groups and categorical variables in Table 1 and Kruskal–Wallis test was used for age at death. Linear regression analysis was used to assess if Braak stage, Thal phase, CERAD score, cortical Aβ burden, or the study groups explained the variation in NFT density in the hippocampal subfields or in the CA2/CA1 and CA2/EC ratio scores. When the study groups were used as predictors, they were dummy coded using PART as the reference group. The results were adjusted for sex and age at death, and also for AGs, LATE‐NC, and LRP when indicated. The maturation process and the correlation between LRP/LATE‐NC and hippocampal subfield NFT densities were analyzed in the same way with linear regression analysis. *P* values < 0.05 were considered significant.

**TABLE 1:** Demographics, neuropathological features, *APOE*, and dementia status of participants.

	All participants^a^	PART	Low ADNC	Moderate ADNC	High ADNC	*p* ^c^
*n* ^b^	210 (100.0)	44 (21.0)	21 (10.0)	71 (33.8)	63 (30.0)	
Sex						
Male	33 (15.7)	9 (20.5)	1 (4.8)	12 (16.9)	8 (12.7)	NS
Female	177 (84.3)	35 (79.5)	20 (95.2)	59 (83.1)	55 (87.3)	
Age at death (median), years	91.5	91.3	91.8	92.4	90.9	NS
Thal phase						
0	13 (6.2)	13(29.5)	0	0	0	NA
1–2	38 (18.1)	31(70.5)	4 (19.0)	1 (1.4)	0	
3	24 (11.4)	0	6 (28.6)	8 (11.3)	1 (1.6)	
4–5	135 (64.3)	0	11 (52.4)	62 (87.3)	62 (98.4)	
CERAD score						
0	55 (26.2)	44(100.0)	0	0	0	NA
1	21 (10.0)	0	21(100.0)	0	0	
2	114 (54.3)	0	0	66 (93.0)	48 (76.2)	
3	20 (9.5)	0	0	5 (7.0)	15 (23.8)	
Braak stage						
I–II	40 (19.0)	17 (38.6)	8 (38.1)	11 (15.5)	0	NA
III–IV	105 (50.0)	27 (61.4)	13 (61.9)	60 (84.5)	0	
V–VI	65 (31.0)	0	0	0	63(100.0)	
LRP						
None	120 (57.1)	30 (68.2)	14 (66.7)	41 (57.7)	29 (46.0)	.030^d^
Olfactory only	8 (3.8)	0	2 (9.5)	0	5 (7.9)	
Amygdala predominant	7 (3.3)	0	0	2 (2.8)	4 (6.3)	
Non‐classifiable	8 (3.8)	0	1 (4.8)	5 (7.0)	1 (1.6)	
Brainstem predominant	15 (7.1)	4 (9.1)	1 (4.8)	6 (8.5)	3 (4.8)	
Limbic	29 (13.8)	7 (15.9)	2 (9.5)	8 (11.3)	11 (17.5)	
Diffuse neocortical	23 (11.0)	3 (6.8)	1 (4.8)	9 (12.7)	10 (15.9)	
LATE‐NC						
0	89 (42.4)	24 (55.8)	13 (65.0)	34 (47.9)	16 (26.2)	.002^e^
1a	19 (9.0)	3 (7.0)	1 (5.0)	6 (8.5)	8 (13.1)	
1b	13 (6.2)	2 (4.7)	1 (5.0)	5 (7.0)	5 (8.2)	
1c	16 (7.6)	4 (9.3)	0	7 (9.9)	4 (6.6)	
2	57 (27.1)	7 (16.3)	4 (20.0)	13 (18.3)	27 (44.3)	
3	11 (5.2)	3 (7.0)	1 (5.0)	6 (8.5)	1 (1.6)	
CAA						
None	58 (27.6)	33 (75.0)	8 (38.1)	13 (19.1)	3 (4.8)	<0.001
Type 1	53 (25.2)	1 (2.3)	0	22 (32.4)	29 (46.0)	
Type 2	96 (45.7)	10 (22.7)	13 (61.9)	33 (48.5)	31 (49.2)	
ARTAG (HC)						
Any	84 (41.8)	16 (37.2)	5 (25.0)	32 (47.8)	28 (46.7)	NS
Subependymal TSA	34 (16.9)	5 (11.6)	3 (15.0)	12 (17.9)	12 (20.0)	NS
Subpial TSA	53 (26.4)	8 (18.6)	5 (25.0)	22 (32.8)	15 (25.0)	NS
Perivascular TSA	36 (17.9)	6 (14.0)	4 (20.0)	11 (16.4)	15 (25.0)	NS
WM TSA	22 (10.9)	4 (9.3)	3 (15.0)	7 (10.4)	7 (11.7)	NS
GM ARTAG	38 (18.9)	10 (23.3)	2 (10.0)	15 (22.4)	9 (15.0)	NS
WM ARTAG	23 (11.4)	5 (11.6)	3 (15.0)	7 (10.4)	7 (11.7)	NS
AGs						
No	156 (74.6)	29 (65.9)	13 (65.0)	52 (73.2)	54 (85.7)	NS
Yes	53 (25.4)	15 (34.1)	7 (35.0)	19 (26.8)	9 (14.3)	(0.059)
*APOE* genotype						
22	1 (0.5)	1 (2.3)	0	0	0	<0.001
23	21 (10.0)	10 (23.3)	0	4 (6.3)	3 (5.2)	
33	114 (54.3)	29 (67.4)	17 (94.4)	40 (62.5)	23 (39.7)	
24	5 (2.4)	1 (2.3)	0	2 (3.1)	2 (3.4)	
34	49 (23.3)	2 (4.7)	1 (5.6)	18 (28.1)	28 (48.3)	
44	2 (1.0)	0	0	0	2 (3.4)	
Dementia						
No	88 (41.9)	26 (59.1)	13 (61.9)	32 (45.1)	10 (15.9)	<0.001
Yes	122 (58.1)	18 (40.9)	8 (38.1)	39 (54.9)	53 (84.1)	

Abbreviations: ADNC, Alzheimer's disease neuropathologic change; AGs, argyrophilic grains; *APOE*, apolipoprotein E; ARTAG, age‐related tau astrogliopathy; CAA, cerebral amyloid angiopathy; CERAD, Consortium to Establish a Registry for Alzheimer's Disease; GM, gray matter; HC, hippocampus; LATE‐NC, limbic‐predominant age‐related TDP‐43 encephalopathy neuropathologic change; LRP, Lewy‐related pathology; NA, not applicable (groups defined by Thal phase, CERAD score, and Braak stage); NS, not significant; PART, primary age‐related tauopathy; TSA, thorn‐shaped astrocytes; WM, white matter.

^a^ Eleven participants (5.2%) were missing from the study groups due to falling outside the grouping criteria (2 participants with CERAD 0, Thal phase 1–2 but Braak stage V–VI and 9 participants with CERAD 0, Thal phase 3, and Braak stage I–IV).

^b^ Data were available for analysis for all participants except for the following variables: LATE‐NC (*n* = 205/210), ARTAG (*n* = 201/210), *APOE* status (*n* = 192/210), CAA (207/210), and AGs (209/210).

^c^ When comparing the groups, Fisher exact test was used for categorical variables, and Kruskal–Wallis test for age at death.

^d^ *P* value for comparing no LRP versus any LRP between PART and high ADNC. There was no statistical significance for LRP between PART and low or moderate ADNC.

^e^ LATE‐NC stages 1a or 2 or 3 versus no LATE‐NC.

All values are *n* (%) unless otherwise indicated.

## 3 RESULTS

### 3.1 Descriptive information on study participants

Frequencies, demographic and neuropathological information, and *APOE* and dementia status of all study participants (*n* = 210) and the PART and ADNC groups are shown in Table 1. The same information for definite and possible PART separately is presented in Table S1 in supporting information. There were no statistically significant differences in sex and age at death between groups. Fisher exact test showed a statistically significant association between the study groups and *APOE* genotype (*p* < 0.001), as shown previously.^12^ Dementia also showed a significant association (Fisher exact test, *p* < 0.001).

### 3.2 NFT densities in hippocampal subfields

**FIGURE 1:**
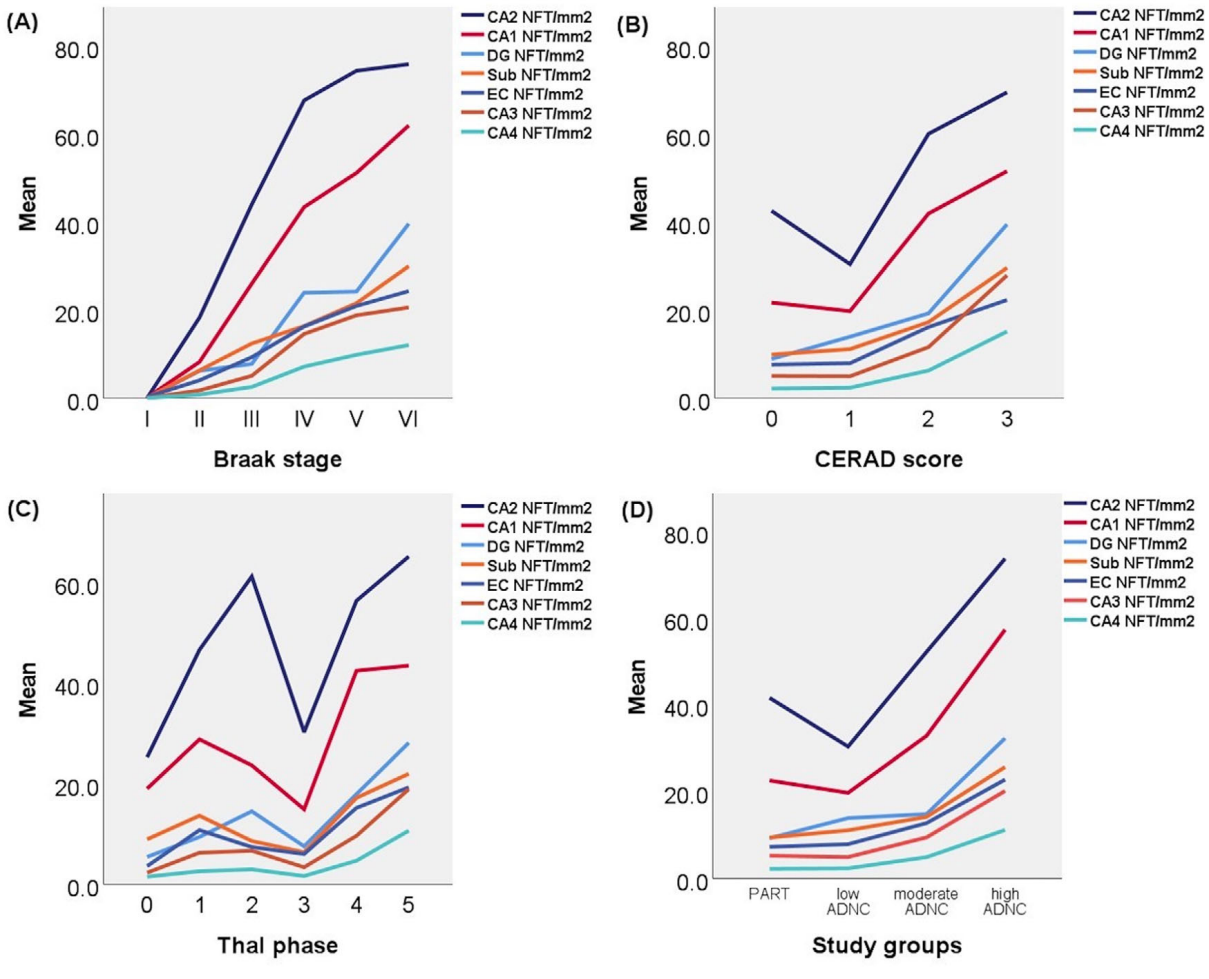
Mean NFT density in each hippocampal subregion across (A) Braak stage, (B) CERAD score, (C) Thal phase, and (D) PART and ADNC groups. ADNC, Alzheimer's disease neuropathologic change; CERAD, Consortium to Establish a Registry for Alzheimer's Disease; DG, dentate gyrus; EC, entorhinal cortex; NFT, neurofibrillary tangle; PART, primary age‐related tauopathy; Sub, subiculum.

Figure 1 shows the average total NFT densities in each hippocampal subfield across different variables. Mean NFT density increased in every hippocampal subfield (EC, CA1–4, subiculum, DG) with increasing Braak stage, CERAD score, and Thal phase (Figure 1A‐C).

**FIGURE 2:**
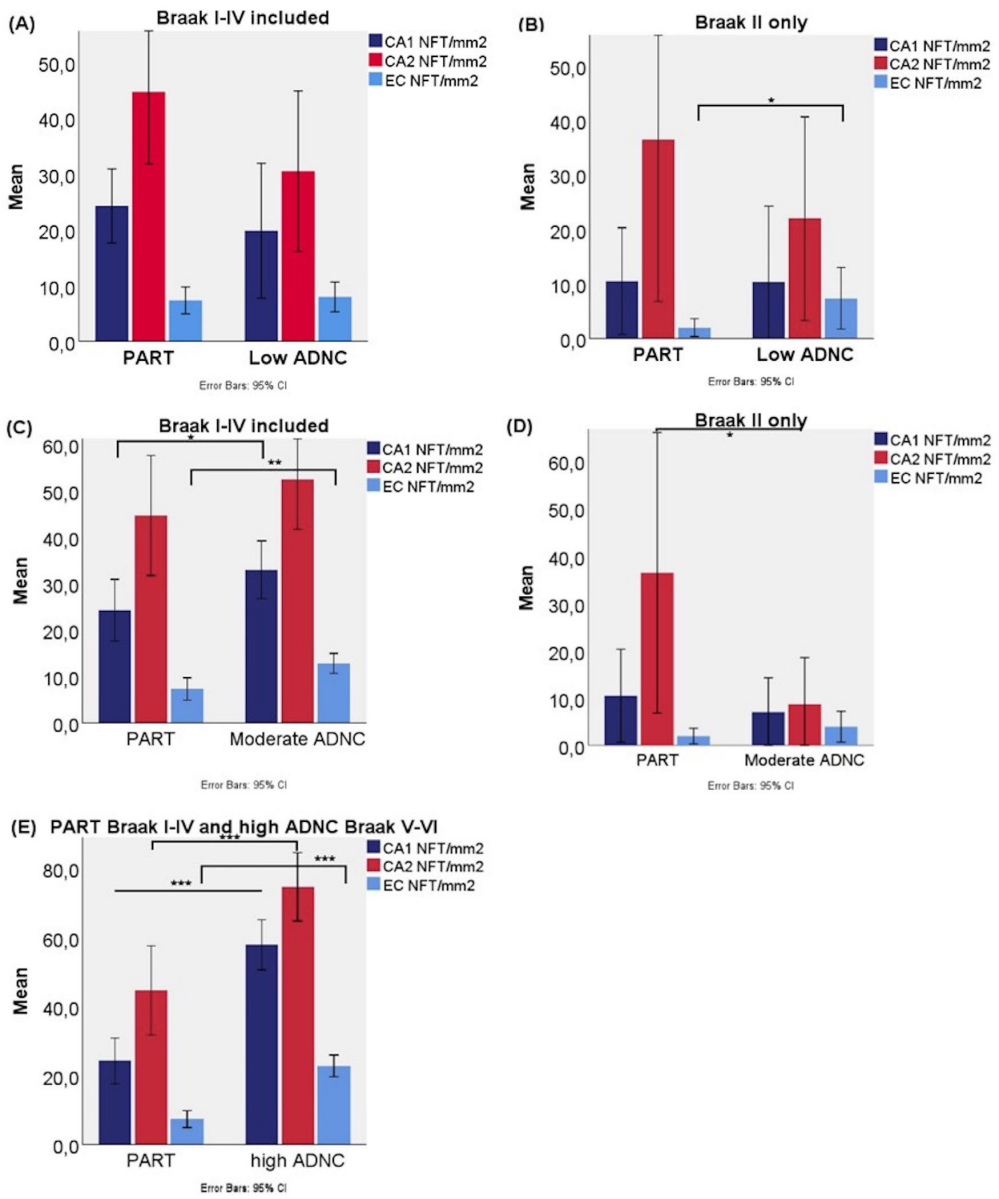
Mean total NFT density in hippocampal subregions CA1, CA2, and EC in PART versus ADNC groups. A, PART versus low ADNC. B, PART (*n* = 16) versus low ADNC (*n* = 7) for Braak stage II only. C, PART versus moderate ADNC. D, PART (*n* = 16) versus moderate ADNC (*n* = 11) for Braak stage II only. E, PART versus high ADNC. *p value < 0.05, **p value < 0.01, ***p value < 0.001. ADNC, Alzheimer's disease neuropathologic change; CI, confidence interval; EC, entorhinal cortex; NFT, neurofibrillary tangle; PART, primary age‐related tauopathy.

In Figure 2, mean total NFT densities in the hippocampal subfields CA1, CA2, and EC are shown comparing the PART and ADNC groups similarly as previously shown by Walker et al. (see Discussion section).^32^, ^33^ Results of statistical analyses are shown in Table S2 in supporting information. There were no statistically significant differences in NFT density in CA1, CA2, and EC between PART and low ADNC (Figure 2A), whereas moderate ADNC showed statistically significantly higher NFT densities in CA1 (*B* = 10.02, *p*  =  0.023) and EC (*B* = 5.90, *p *= 0.002) than PART but not CA2 (Figure 2C). As expected, high ADNC showed statistically significantly higher NFT densities in CA1, CA2, and EC compared to PART (Figure 2E; *B* = 32.91, *B* = 25.60, and *B* = 16.04, respectively, and *p *< 0.001). Because early disease stage was of interest, we next analyzed individuals with Braak stage II and compared PART to low and moderate ADNC (Figure 2B and 2D). The results of these statistical analyses are shown in Table S3 in supporting information. Low ADNC showed statistically significantly higher NFT density in EC compared to PART (Figure 2B; *B* = 5.10, *p*  =  0.018), but there were no statistically significant differences when analyzing the CA1 and CA2 subregions. Moderate ADNC showed significantly higher NFT density in CA2 compared to PART (Figure 2D; *B* = –31.37, *p*  =  0.014). There were no statistically significant differences with CA1 and EC. When Braak III and IV were analyzed separately, no statistically significant differences were found between the PART and low and moderate ADNC groups.

For the remaining hippocampal subfields (Figure 1D), CA3, CA4, subiculum, and DG showed no statistically significant differences between PART and low ADNC in the age‐ and sex‐adjusted model. Moderate ADNC showed higher NFT densities than PART in subiculum (*B*  =  5.32, *p*  =  0.021, age‐ and sex‐adjusted), but not in CA3 (*p* = 0.073), CA4 (*p* = 0.059), or the DG (*p* = 0.265). High ADNC had significantly higher NFT densities in CA3, CA4, subiculum, and DG than in PART (*B* = 12.85, *B* = 8.19, *B* = 16.72, and *B* = 26.57, respectively, and *p* < 0.001).

**FIGURE 3:**
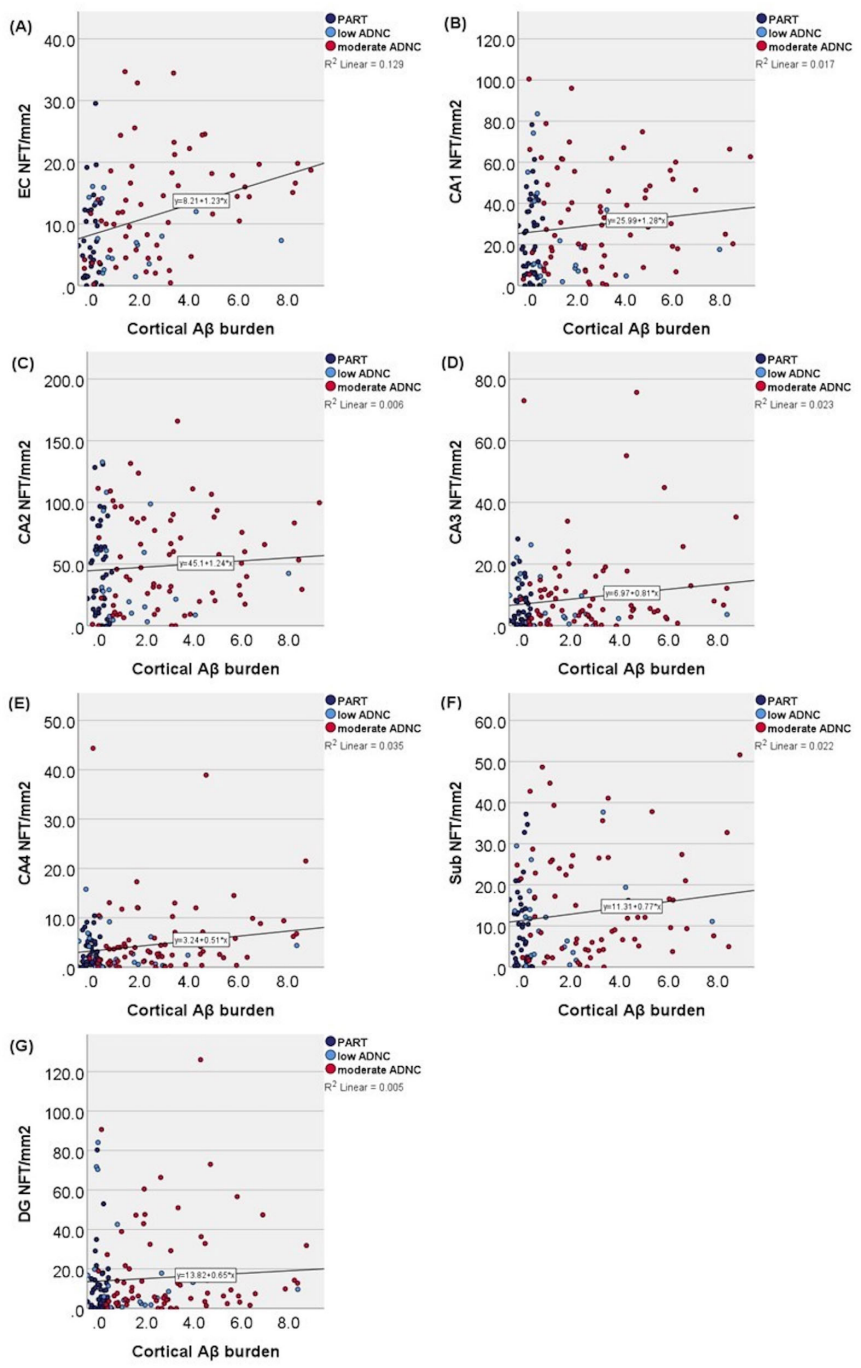
Correlation between cortical Aβ burden and NFT density in each hippocampal subfield in Braak I through IV individuals. Results of statistical analyses for the (A) EC: *B*  =  1.28, 95% CI 0.69 to 1.88, *p *< 0.001; (B) CA1 subfield: *B*  =  1.54, 95% CI –0.13 to 3.20, *p *= 0.071*; (C) CA2 subfield: NS (*p* = 0.256); (D) CA3 subfield: *B*  =  0.89, 95% CI 0.03 to 1.75, *p *= 0.042; (E) CA4 subfield: *B*  =  0.55, 95% CI 0.12 to 0.98, *p *= 0.013; (F) subiculum: *B*  =  –0.85, 95% CI –0.05 to 1.75, *p *= 0.065*; and (G) DG: NS (*p* = 0.289). *Borderline significance. Aβ, amyloid beta; ADNC, Alzheimer's disease neuropathologic change; CI, confidence interval; DG, dentate gyrus; EC, entorhinal cortex; NFT, neurofibrillary tangle; NS, not significant; PART, primary age‐related tauopathy.

Figure 3 shows how cortical Aβ burden correlated with NFT density in each hippocampal subfield. As early disease stage was of interest, only Braak I through IV individuals were included in this analysis to avoid end‐stage AD influencing the results. Table S4 in supporting information shows the results of statistical analyses. In the unadjusted analysis, the EC (Figure 3A; *B*  =  1.28, *p *< 0.001), CA3 (Figure 3D; *B*  =  0.89, *p *= 0.042), and CA4 (Figure 3E; *B*  =  0.55, *p *= 0.013) subfields showed a statistically significant positive correlation between increasing cortical Aβ burden and NFT density. CA1 (Figure 3B; *p *= 0.071) and subiculum (Figure 3F; *p *= 0.065) showed a positive correlation between increasing cortical Aβ burden and NFT density with borderline significance. CA2 (Figure 3C; *p* = 0.256) and DG (Figure 3G; *p* = 0.289) did not show a statistically significant correlation between the cortical Aβ burden and NFT density. Adjusting for age and sex showed similar results (Table S4).

### 3.3 Analysis of the CA2/CA1 NFT ratio

**FIGURE 4:**
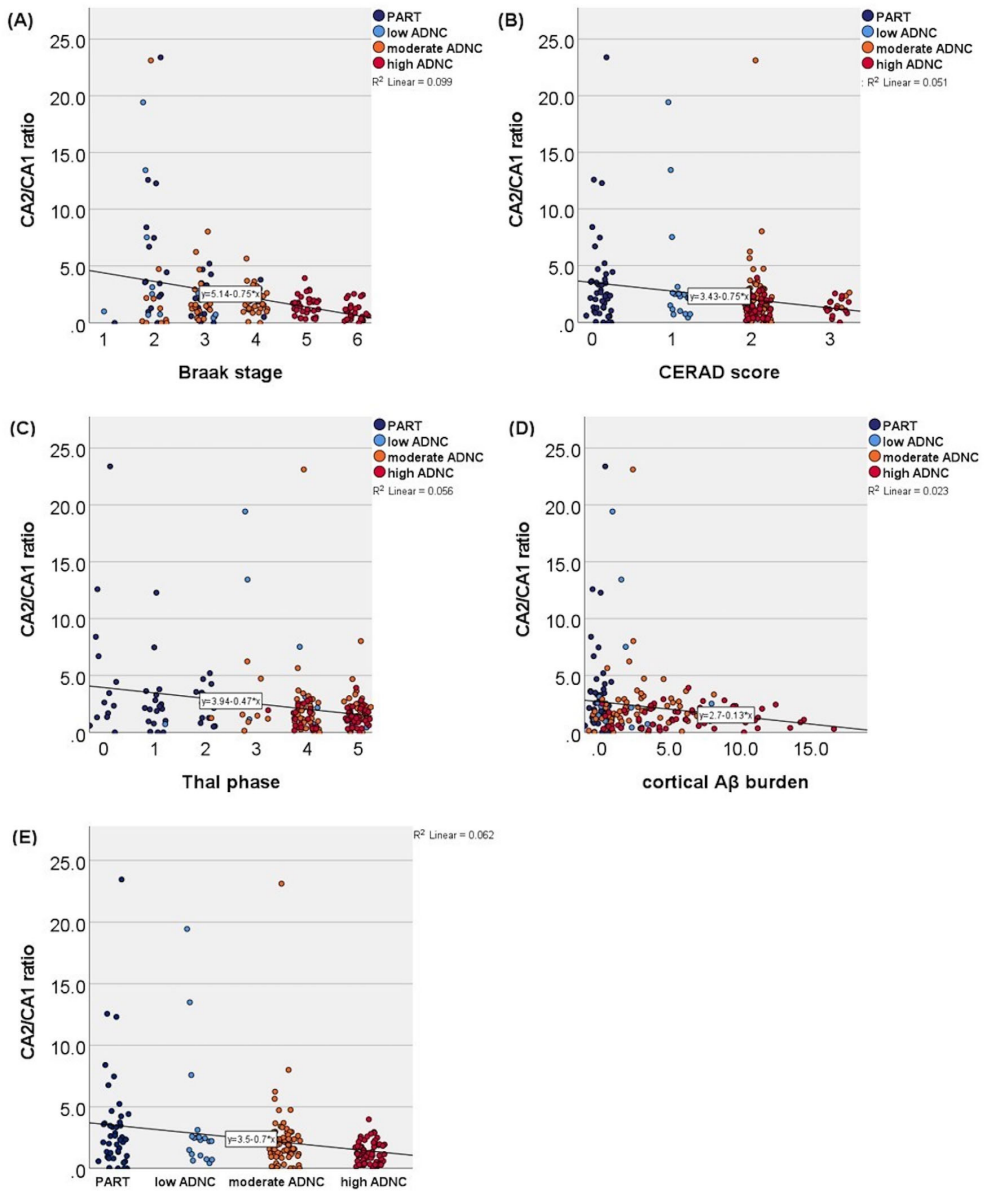
The CA2/CA1 ratio showed an inverse association with (A) Braak stage (*B*  =  –0.79, 95% CI –1.13 to –0.44, *p *< 0.001), (B) CERAD score (*B*  =  –0.75, 95% CI –1.21 to –0.29, *p *= 0.001), (C) Thal phase (*B*  = –0.44, 95% CI –0.73 to –0.15, *p *= 0.003), and (D) cortical Aβ burden (*B*  =  –0.15, 95% CI –0.27 to –0.02, *p *= 0.020). E, Distribution of CA2/CA1 ratios across PART and ADNC groups. Aβ, amyloid beta; ADNC, Alzheimer's disease neuropathologic change; CERAD, Consortium to Establish a Registry for Alzheimer's Disease; CI, confidence interval; DG, dentate gyrus; EC, entorhinal cortex; NFT, neurofibrillary tangle; PART, primary age‐related tauopathy.

**FIGURE 5:**
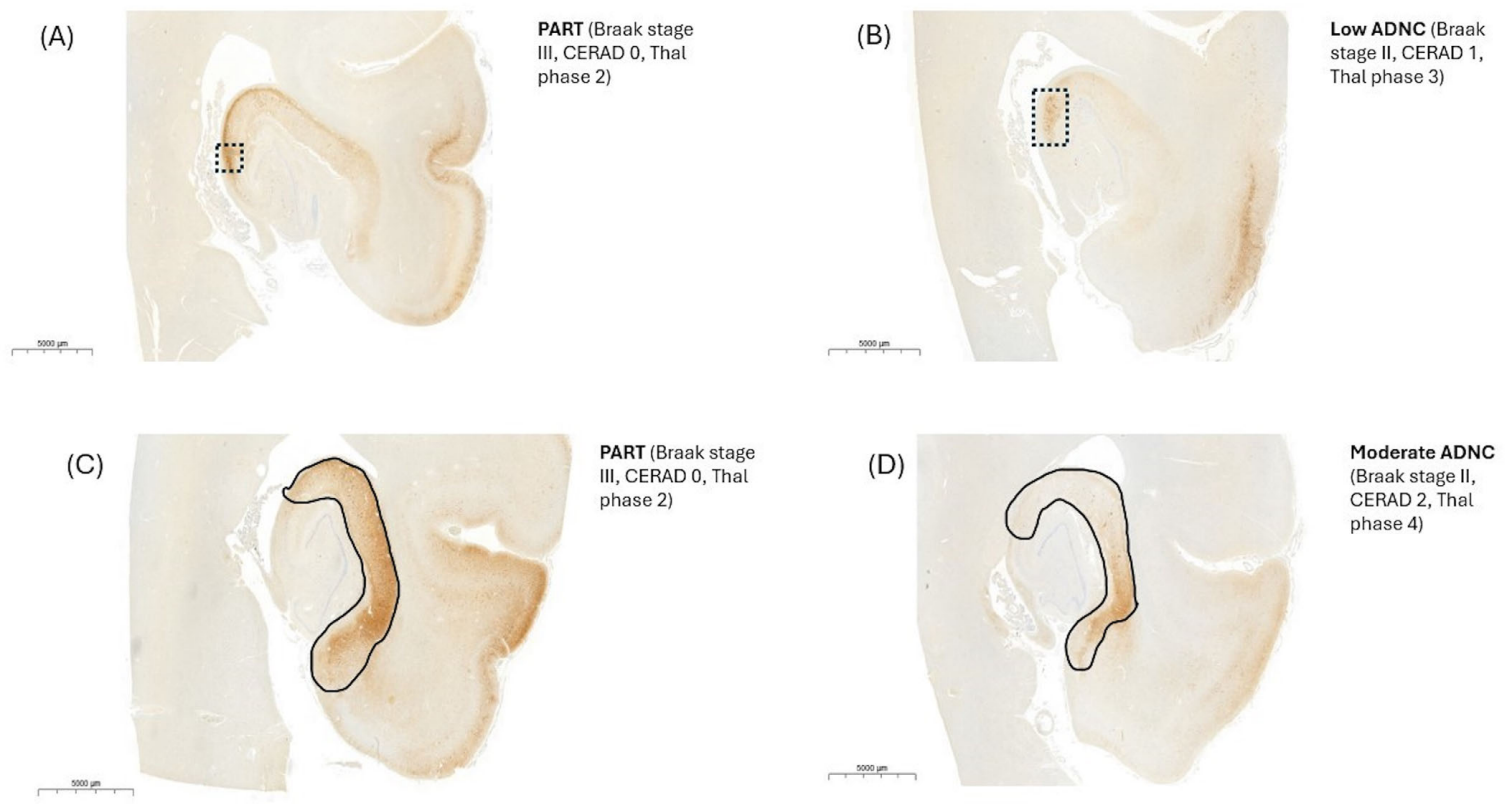
Representative AT8‐stained hippocampal micrographs showing (A) CA2 conspicuous pattern (box) in a participant with PART (Braak stage III, CERAD 0, Thal phase 2, CA2/CA1 ratio 4.7); (B) CA2 conspicuous pattern (box) in a participant with with low ADNC (Braak stage II, CERAD 1, Thal phase 3, CA2/CA1 ratio 12.5). (C) CA1/subiculum‐predominant pattern (marked) in a participant with PART (Braak stage III, CERAD 0, Thal phase 2, CA2/CA1 ratio 0.3). (D) CA1/subiculum‐predominant pattern (marked) in a participant with moderate ADNC (Braak stage II, CERAD 2, Thal phase 4, CA2/CA1 ratio 0.3). ADNC, Alzheimer's disease neuropathologic change; CERAD, Consortium to Establish a Registry for Alzheimer's Disease; PART, primary age‐related tauopathy.

Next, we examined the CA2/CA1 NFT ratio in accordance with Walker et al.^32^, ^33^ Figure 4 shows the CA2/CA1 ratio across Braak stage, CERAD score, Thal phase, cortical Aβ burden, and PART and ADNC groups. The CA2/CA1 ratio showed an inverse association with Braak stage (Figure 4A; *B* = –0.79, *p *< 0.001), CERAD score (Figure 4B; *B* = –0.75, *p *= 0.001), Thal phase (Figure 4C; *B* = –0.75, *p *= 0.003), and cortical Aβ burden (Figure 4D; *B* = –0.15, *p *= 0.020). Comparing PART to the ADNC groups (Figure 4E, Table S5 in supporting information), there was no statistically significant difference in CA2/CA1 ratios between PART and low ADNC. Comparing PART and moderate ADNC, there was a difference, with borderline significance (Figure 4E; *B* = –1.16, *p* = 0.053). High ADNC showed statistically significantly lower CA2/CA1 ratios than PART (Figure 4E; *B* = –2.04, *p *= 0.001). Representative images of study individuals on both the higher and lower end of CA2/CA1 values are shown in Figure 5, visualizing a CA2 versus CA1/subiculum conspicuous pattern of neurofibrillary degeneration.

The CA2/CA1 ratio had several extreme outlier values that corresponded approximately to the 90th percentile of values. Table S6 in supporting information shows the features of these individuals compared to all individuals below the 90th percentile. All CA2/CA1 outliers had Braak stage II and III except for one individual with Braak stage IV. Most had CERAD score 0 and most were also PART individuals. Thal phases were evenly distributed between the outlier individuals. Only one outlier had a genotype containing the *APOE* ε4 allele. In other words, they showed more “PART‐like” than “AD‐like” features.

In addition to the CA2/CA1 ratio, we also analyzed the CA2/EC NFT ratio and found that the Braak stages (Figure S3A in supporting information; *B* = –6.08, *p *= 0.044) , CERAD scores (Figure S3B; *B* = –9.93, *p *= 0.014), and Thal phases (Figure S3C; *B* = –6.97, *p *= 0.007) were also inversely associated with the CA2/EC ratio. Moderate ADNC (*B* = –28.26, *p *= 0.012) and high ADNC (*B* = –27.38, *p *= 0.021) both had statistically significantly lower CA2/EC ratios compared to PART (Figure S3D). For cortical Aβ burden the result was not statistically significant (Figure S3E).

### 3.4 Maturation process of NFTs in PART and ADNC

**FIGURE 6:**
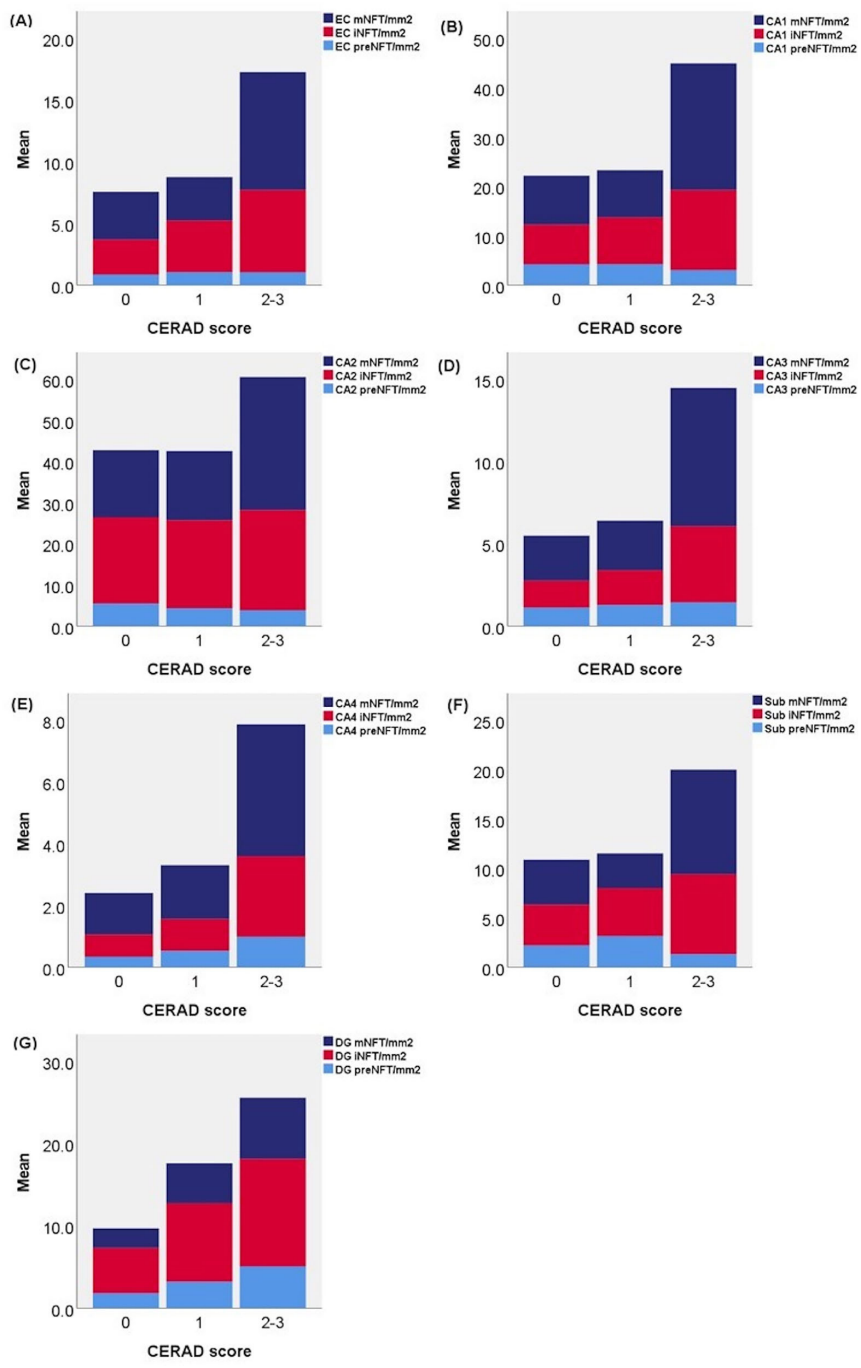
NFT maturity levels (mean preNFT, iNFT, and mNFT density) across CERAD scores in the (A) EC, (B) CA1 subfield, (C) CA2 subfield, (D) CA3 subfield, (E) CA4 subfield, (F) subiculum, and (G) DG. CERAD, Consortium to Establish a Registry for Alzheimer's Disease; DG, dentate gyrus; EC, entorhinal cortex; iNFT, intermediary neurofibrillary tangle; mNFT, mature neurofibrillary tangle; NFT, neurofibrillary tangle; preNFT, pre‐neurofibrillary tangle.

Figure 6 shows the NFT maturity levels (preNFT, iNFT, and mNFT density) in relation to CERAD score in every hippocampal subfield. Table S7 in supporting information shows results of statistical analyses. There were no statistically significant differences in preNFT, iNFT, and mNFT densities in any hippocampal subfield comparing CERAD score 0 and 1. Comparing CERAD score 0 and 2 to 3, the latter showed statistically significantly higher mNFT densities in all hippocampal subfields, whereas iNFT densities were statistically significantly higher in all hippocampal subfields except for CA2 (Figure 6C). PreNFT densities showed either no difference or statistically significantly higher or even lower scores in CERAD 2 and 3 compared to CERAD 0. CA2 was distinguished from the other subfields as it was the only subfield in which the preNFT and iNFT density scores were similar in CERAD score 0 versus CERAD score 2 and 3 (Figure 6C). The NFT maturity levels in relation to Thal phases are shown in Figure S4 in supporting information, and results of statistical analyses are shown in Table S8 in supporting information. Thal data correlated similarly but less consistently with preNFT, iNFT, and mNFT densities than the CERAD score, reflecting the sensitive nature of the Thal method, as this score includes scarce diffuse plaques which might have only a minimal impact on NFTs.

**FIGURE 7:**
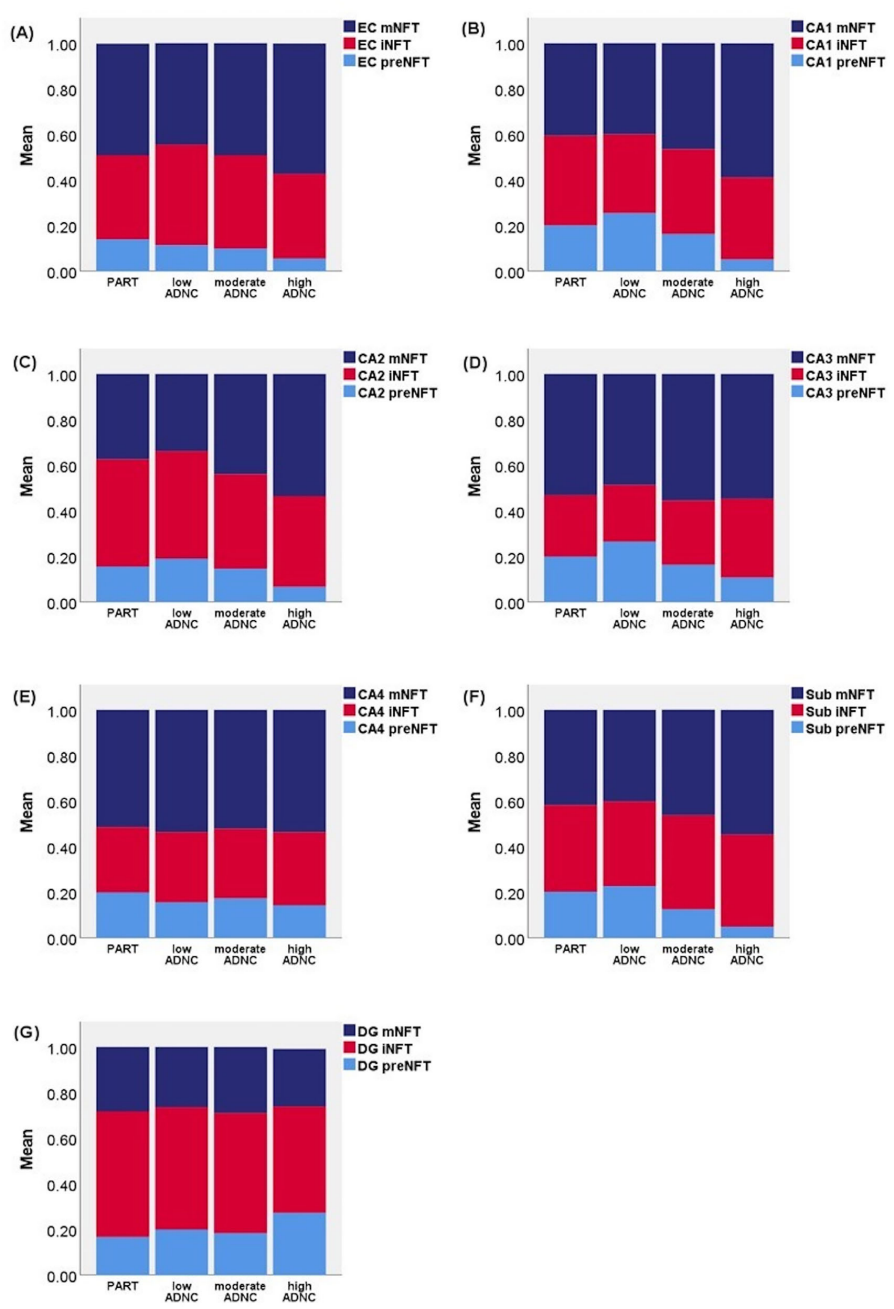
Proportion of NFT maturity levels (preNFT, iNFT, mNFT) across PART and ADNC groups in the (A) EC, (B) CA1 subfield, (C) CA2 subfield, (D) CA3 subfield, (E) CA4 subfield, (F) subiculum, and (G) DG. ADNC, Alzheimer's disease neuropathologic change; DG, dentate gyrus; EC, entorhinal cortex; iNFT, intermediary neurofibrillary tangle; mNFT, mature neurofibrillary tangle; NFT, neurofibrillary tangle; preNFT, pre‐neurofibrillary tangle.

Figure 7 shows the NFT maturity levels expressed as proportions of total NFTs compared between the PART and ADNC groups and separately for each hippocampal subfield. Results of statistical analyses are shown in Table S9 in supporting information. In most hippocampal subfields, the proportion of mNFTs increased and the proportion of preNFTs decreased when moving from PART toward high ADNC (Figure 7A‐F), which appears logical. DG showed the converse result for preNFTs, as high ADNC had a statistically significantly larger preNFT proportion than PART (Figure 7G).

### 3.5 Adjusting for comorbidities

Selective neurofibrillary degeneration in CA2 is a distinguishing feature of the 4‐repeat (4R) tauopathies, particularly argyrophilic grain disease (AGD).^51^ According to the current PART classification criteria, other NFT‐associated diseases should be absent for a PART diagnosis to be made; however, the presence of AGs is allowed. AGs were very common in the Vantaa 85+ Study (≈ 25%), and comorbidity of AGD is therefore a possible confounding factor for our results. When analyzed on their own, AGs showed a strong independent association with preNFTs in every hippocampal subfield and with iNFTs in the subiculum (*B*‐value ranging from 0.87 to 6.25, *p* ≤ 0.001, age‐ and sex‐adjusted analysis). AGs were more common in PART cases than in high AD cases (Table 1), but among PART cases AGs were found to be more common in subjects with higher Braak stages (Table S10 in supporting information). The effects of adding AGs as a covariate to our regression models is shown in Tables S2–S5 and S7–S9. To summarize, the statistical significances of Figures 2, 3, and 4 remained and some even strengthened. When adjusting the statistical model of Figure 6 for AGs, the main effect was that the statistically significantly lower preNFT density scores (in CA1 and subiculum) in CERAD 2 and 3 compared to 0 was annulled. Adjusting the data in Figure 7 only had a minor effect on the trend that mNFT proportions increased and preNFT proportions decreased when moving from PART toward high ADNC.

Other possible confounders of interest were LRP and LATE‐NC. Figure S5 in supporting information shows how the mean NFT density in each hippocampal subfield correlated with LRP type, LATE‐NC stage, and AGs. When analyzed on their own, the only statistically significant result between LRP type and hippocampal subfield NFT density was a positive association between subicular NFT density and amygdala‐predominant LRP type (Figure S5A; *B* = 12.5, *p *= 0.017, age‐ and sex‐adjusted linear regression analysis). LRP has been shown to appear in the CA2 and 3 subfield of the hippocampus in DLB,^52^, ^53^ but in the present study, we could not find a statistically significant association between semiquantitatively assessed LRP in CA2 and 3^44^ and NFT density in CA2. Adjusting the statistical models of Figures 2, 4, and 6, 7 for LRP made no noteworthy difference in the statistical significance levels. Adjusting the results in Figure 3 for LRP weakened the association between cortical Aβ burden and hippocampal NFT densities, leaving only EC and CA4 statistically significant and CA3 borderline significant (Table S4).

The LATE‐NC stages 1a, 2, and 3, compared to stage 0, showed a statistically significant positive association with NFT density in all hippocampal subfields (Figure S5B; *B*‐value ranging from 3.84 to 16.21, *P* value ranging from *p* < 0.001 to *p *= 0.044, age‐ and sex‐adjusted linear regression analysis). Adjusting the statistical models of Figures 2, 4, and 7 for LATE‐NC made no noteworthy difference in the statistical significance levels. Adjusting the results in Figure 3 with LATE‐NC weakened the association between cortical Aβ burden and hippocampal NFT densities, leaving only EC and CA4 statistically significant (Table S4). Adjusting the data in Figure 7 with LATE‐NC had minor effects on the results (Table S9).

### 3.6 Drivers of cognitive decline in PART

Next, we looked at what driving factors, if any, were associated with cognitive decline in the PART individuals. Table S10 shows the cognitive status, *APOE* genotype, and neuropathological features of the PART participants across Braak stages I through IV, and Table S11 shows the results of regression analyses to determine predictors of dementia and the last Mini‐Mental State Examination (MMSE) before death in PART. Possible factors affecting disease severity (i.e., higher Braak stages) in PART are discussed in the supporting information. Braak stage, LRP, LATE‐NC, CAA, AGs, or ARTAG did not associate with dementia or the last MMSE before death in our PART participants. Arteriolosclerosis in the frontal white matter associated with dementia (odds ratio = 2.73, *p* = 0.009). NFT density in CA1 (*B* = –0.17, *p* = 0.039) showed an inverse association with the last MMSE before death. The NFT densities in the other hippocampal subfields did not associate with either dementia or the last MMSE before death.

## 4 DISCUSSION

The primary objective of this study was to investigate if and how hippocampal distribution of NFTs is affected by Aβ deposition in elderly individuals from the population‐based Vantaa 85+ Study. A population‐based sample allowed us to examine the whole spectrum of Aβ and neurofibrillary changes in the elderly population, making it an ideal setting to explore the finding of differing hippocampal distribution of neurofibrillary degeneration in PART and AD observed in hospital‐based studies. Here, we found that moderate ADNC had significantly higher NFT density in EC, CA1, and subiculum but not in CA2 compared to PART. In Braak stage II, NFT density was lower in CA2 in moderate ADNC and higher in EC in low ADNC compared to PART. No other significant differences were observed between low ADNC and PART. High ADNC had higher NFT density in all hippocampal subfields compared to PART.

CA2/CA1 and CA2/EC NFT ratios were inversely associated with Aβ deposition and with PART versus moderate and high ADNC. NFT density in each hippocampal subregion except CA2 and DG correlated statistically significantly or borderline significantly with cortical Aβ burden. Adjusting for AGs did not change these results. Adjusting for LRP and LATE‐NC reduced the association between hippocampal NFT densities and cortical Aβ burden, which might be mediated through their association with AD.^43^, ^44^

Walker et al. first presented the hypothesis that PART shows an early vulnerability to neurofibrillary degeneration in the hippocampal CA2 subfield, whereas AD initially has more severe neurofibrillary degeneration in the EC and CA1, relatively sparing CA2 until later in the disease process.^32^, ^33^ Key findings from their studies include the following: intermediate‐to‐high ADNC showed significantly higher neurofibrillary pathology in CA1 compared to PART, but no significant difference in CA2;^32^, ^33^ neurofibrillary degeneration in all examined hippocampal subfields except CA2 correlated significantly with increasing Aβ burden;^33^ and CA2/CA1 ratios of neurofibrillary degeneration were inversely associated with Aβ burden^33^ and with PART versus intermediate‐to‐high ADNC.^32^, ^33^ The results of the present study, as summarized above, are consistent with these findings. A difference between our studies was that Walker et al. included only a few ADNC cases relative to PART cases. Including a wider unselected ADNC spectrum in the present study highlighted the heterogenicity of the CA2/CA1 ratio. In addition, Walker et al. combined intermediate‐to‐high ADNC cases, whereas we chose to separate moderate and high ADNC so that the observed differences would not be driven by end‐stage AD. Still, we confirm that PART cases can show a predisposition for neurofibrillary degeneration in CA2 compared to moderate ADNC.

A small Japanese study^54^ on hippocampal regional distribution of NFTs in non‐demented elderly individuals with Braak stages II and III identified two patterns, namely CA2‐predominant and subiculum/preCA1‐predominant. These individuals had Braak senile plaque (SP) stages 0‐B with no significant difference between the patterns. Most late‐onset AD brains (history of dementia, Braak stages III–IV, SP stage C) showed a subiculum/preCA1‐predominant pattern. Unlike this study, our findings did show a correlation between CA2 predominance and lower Aβ burden at early disease stage. The Japanese study's small sample size (*n* = 45) or different Aβ staging system might explain the discrepancy. In the present study, we showed that higher CA2/CA1 ratios were not limited to CERAD 0 and Thal 0. Together, our studies suggest that the CA2‐predominant pattern is not exclusive to Aβ‐negative cases, but the subiculum/CA1‐predominant pattern is more predictive of AD.

The proposition that PART shows an early predisposition for neurofibrillary degeneration in CA2 was recently questioned by Del Tredici et al.^25^ In their study, which consisted of 325 definite PART cases with a mean age of 66.5 years (range 28–100), CA1 had a tau rating that was greater than or equal to that of CA2 in 89% of cases. The remaining cases did show a predilection for neurofibrillary degeneration in CA2, but most were deemed to be dependent on the level of the hippocampal formation being assessed. Only 4% were thought to have a true CA2 focus of neurofibrillary degeneration. Interestingly, the authors suggested that the early involvement of CA2 to neurofibrillary degeneration could be due to projections from subcortical nuclei, especially locus coeruleus.^25^ One possible explanation for the more modest finding of CA2 vulnerability to neurofibrillary degeneration of their study compared to the present study could be the difference in age (mean 66.5 vs. 91.5); as neurofibrillary pathology accumulates with increasing age, the differences between hippocampal subfields might become more apparent in oldest‐old subjects. Our methods also differed (e.g., using 100 µm vs. 4 µm AT8‐stained sections).

The NFTs in PART have been described as “indistinguishable” from those in AD,^7^ as both show the same 3R/4R tau isoforms, are composed of tau‐paired helical filaments, and share an identical fold structure of tau filaments.^7^, ^55^, ^56^, ^57^ However, a recent study found that neocortical tau seeding and post‐translational modification profiles differentiated ADNC from PART.^58^ To further elaborate on differences or similarities in the tauopathy between PART and ADNC, we compared the maturation process of NFTs between them. We found that the density of iNFTs and mNFTs increased in the hippocampus with increasing CERAD neuritic plaque scores, and the proportion of mNFTs increased while the proportion of preNFTs decreased when moving from PART toward high ADNC. This finding is logical as Aβ is thought to accelerate tangle spread.^59^ Our results suggest that the maturation process of hippocampal NFTs occurs sequentially from preNFTs to iNFTs to mNFTs in both PART and ADNC, but the maturation process in the latter is exacerbated by Aβ. Interestingly, the CA2 subregion showed a subtle difference in the maturation process compared to the other hippocampal subfields, as CA2 was the only subfield in which only the mNFT density, but not the preNFT and iNFT densities, was significantly increased between CERAD score 2 and 3 versus 0. This could be an additional implication that neurofibrillary degeneration in the CA2 subfield occurs differently in subjects who do not have cortical neuritic plaques. Additional studies are warranted to further characterize the distinguishing features of NFTs in ADNC versus PART.

A previous study by Iida et al.^60^ reported that quantitative assessment of hyperphosphorylated tau was a better predictor of cognitive decline in PART than the Braak neuroanatomical staging system, and that a quantitative measure is a better way to measure disease severity in PART. Cerebrovascular disease was also a strong predictor of cognitive decline in PART in their cohort.^60^ Our results suggest the same, as CA1 NFT density and arteriolosclerosis in the frontal white matter predicted cognitive decline in our PART individuals. Unlike Iida et al., we could not find an association between ARTAG and cognitive decline in PART.^60^

The main strength of our study material is that it is population based, therefore minimizing the risk of selection bias. It also represents the oldest‐old in which a plateau of Aβ accumulation is reached, making it ideal for researching PART. Unfortunately, many cases had to be excluded to ensure adequate quality and comparability of sections. Although reduced sample size weakens the statistical power, the data were strong enough to obtain significant results. Additionally, we used AI‐derived data, which is arguably a more objective method than semiquantitative analysis and is a more advanced technology than image analysis–based quantitative pixel assessments used in previous studies. Our study passed the Aiforia validation process, in which the AI model is required to perform better than or equal to the average of the human validators (validation figures for our AI model are provided in the supporting information). A weakness of our study is that we only had quantitative neocortical Aβ data based on methenamine silver staining available, but no quantitative Aβ data from the hippocampus. It would have been optimal to analyze quantitative hippocampal Aβ burden similarly to Walker et al.^33^ Still, our data showed a similar trend regarding the correlation between hyperphosphorylated tau in different hippocampal subfields and Aβ burden. Another issue that should be taken into account when viewing the results is that true PART is hard to define. Individuals currently classified as possible PART^7^ (i.e., either with Thal phase 1–2 or CERAD score 1) represent an interface and it is not known if they will evolve into AD. For example, our low ADNC subjects could according to the PART criteria^7^ also be interpreted as possible PART (CERAD score 1). The 9 Thal 3/CERAD 0 individuals that were excluded due to classification challenges are discussed in the supporting information.

In conclusion, we found that a CA2‐conspicuous pattern of hippocampal neurofibrillary degeneration was more likely to be seen with lower Aβ burden (i.e., it was a more “PART‐like” than “AD‐like” feature). However, this was not an exclusive finding, as some ADNC individuals also displayed CA2 conspicuousness relative to CA1. More evidence from unselected cohorts is needed before CA2 predominance can be used as a neuropathological marker for PART. In addition, the mechanisms for how Aβ can alter the distribution of hippocampal neurofibrillary pathology remain unclear and more research on this subject is warranted.

## CONFLICT OF INTEREST STATEMENT

H.P. has provided paid pathology consulting services for Aiforia Technologies Oyj in unrelated projects. All other authors declare no conflicts of interest. Author disclosures are available in the supporting information.

## CONSENT STATEMENT

The study was performed in accordance with the ethical standards defined by the Declaration of Helsinki. The ethics committee of the Health Centre of the City of Vantaa and the ethics committee of the Helsinki and Uusimaa Hospital District approved the study. Access to health and social work records and death certificates were permitted by the Finnish Health and Social Ministry and the National Authority for Medicolegal Affairs (VALVIRA) approved the collection of the tissue samples and their use for research. Informed consent was obtained from all individual participants or their relatives. This study is part of the population‐based Vantaa 85+ Study that included all individuals living in the city of Vantaa aged 85 years or over. The study encompasses the entire population of this demographic group in Vantaa and is inclusive in nature. Therefore, issues related to diversity, equity, and inclusion (DEI) do not apply.

## Supporting information



Supporting information

Supporting information
