# Supplementary material for: Hippocampal tau distribution in primary age‐related tauopathy: The Vantaa 85+ Study
Source: Alzheimers Dement. 2025 Aug 22;21(8):e70613. doi: 10.1002/alz.70613 (PMC12371459; doi:10.1002/alz.70613)
Supplement: Supplementary file 1 — Supporting information [file ALZ-21-e70613-s002.docx]

**Supplementary Material**

**1 Supplementary Methods**

- 1. **Evaluation of ARTAG**

Aging-related tau astrogliopathy (ARTAG) was evaluated in the hippocampus at the level of the lateral geniculate nucleus on AT8-stained slides. According to a previously published evaluation strategy [1], we assessed the presence or absence of thorn-shaped astrocytes (TSA) in subependymal, subpial, perivascular and white matter (WM) location. We also assessed overall WM, grey matter (GM) and any ARTAG, which included granular/fuzzy astrocytes in addition to the TSA.

**1.2 Classification criteria for pretangles, intermediary tangles, and mature tangles**

The classification criteria are based on a publication by Moloney et al [2]. Neurofibrillary tangles (NFT) were divided into the following three classes based on maturity: pretangles, intermediary NFTs, and mature NFTs. Pretangles were defined as neurons containing fine granular AT8-positive staining in the cytoplasm with or without perinuclear staining. Intermediary NFTs were defined as neurons containing more intensely stained and coarser aggregates of tau, possibly with focal darkly stained fibrillar tau accumulation. Mature NFTs were defined as neurons with darkly stained fibrillar tau pathology that filled at least half of the cell. Neurons with fibrillar tau filling the whole cell and lacking a nucleus were included in this category.

**1.3 Training of the artificial intelligence model**

Aiforia Create Version 5.5 (Aiforia Technologies Plc) was used to create an artificial intelligence (AI) model that identified NFTs in three different maturity classes. Hippocampal whole slide images (WSI) from 36 individuals were used as training material. The AI model was first trained to detect high-quality tissue areas under which the pretangle, intermediary NFT, and mature NFT detections were trained (Supplementary Figure 1). Tissue layer was trained using region type detection (semantic segmentation) with “Very complex” neural network complexity at a 789.6-µm field of view. The NFTs layer was trained using object type detection (object detector) with “Extra complex” network complexity using a 17-µm diameter for the object detection for all the three classes (pretangle, intermediary NFT, mature NFT). Image augmentation was changed for object detection layer by 20% for scale, 20% for aspect ratio and maximum shear, 20% for luminescence and contrast, 20% for maximum white balance, and 5% for noise. No change from default was made for image augmentation parameters for the tissue layer. Training was performed with a pre-set value of 7000 iterations for both layers, where 2799 iterations were executed with a final training loss of 0.0025 for the tissue layer and 7000 iterations were executed with a Training loss of 0.2399 for the pretangle, intermediary NFT, and mature NFT classes. In total, 1763 training regions were used and verifications were performed to yield an error of 0.18% for tissue layer (F1 Score; 99.90%) and 0.75% for pretangle, intermediary NFT, and mature NFT layer (F1 Score; 99.63%). To validate the final AI model, three board-certified pathologists (LM, OT, HP) scored NFTs in 25 WSIs separate from the training WSIs. The scoring guidelines for pretangles, intermediary NFTs, and mature NFTs were the same as described earlier. Altogether, 78 validation regions were scored. The results of the validation process (i.e., comparison of human NFT scores with AI model scores) were the following: for each validator, the error for the tissue layer was 0.06%, 0.21%, and 0.06%, and the error for the pretangle, intermediary NFT, and mature NFT layer was 3.57%, 7.86%, and 6.43%, respectively. Morphometric analysis was not enabled for the AI model prior to the image analysis run. Image analysis was performed in Aiforia Hub for specific regions of interest (ROI) in multiple batches. Data were visually monitored and generated using Microsoft Excel.


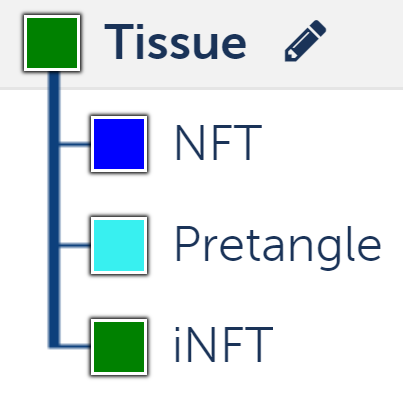
 
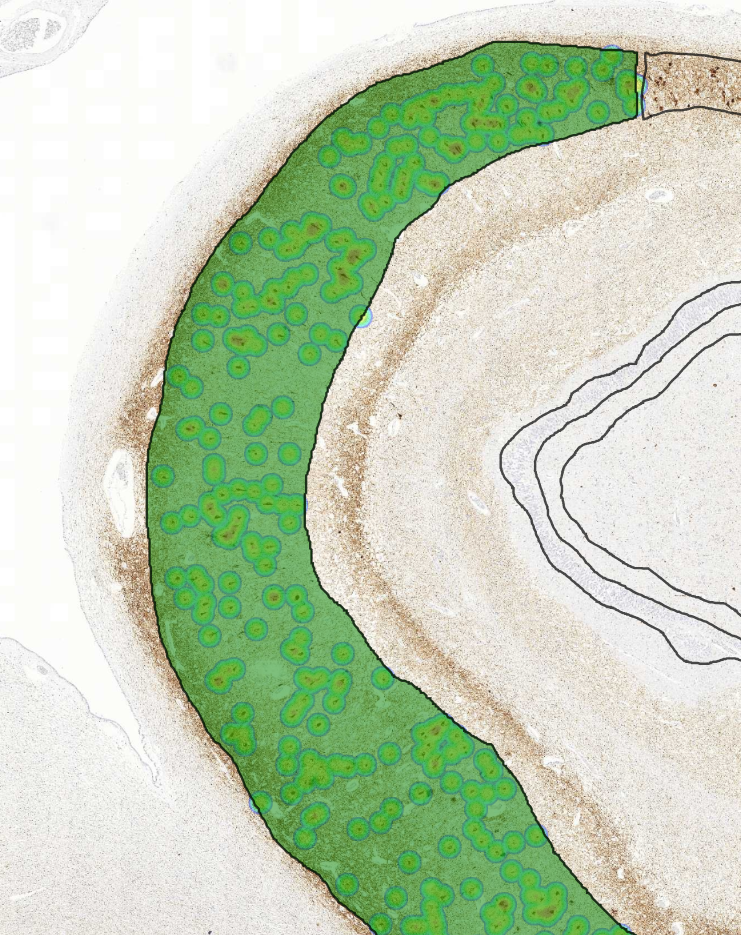

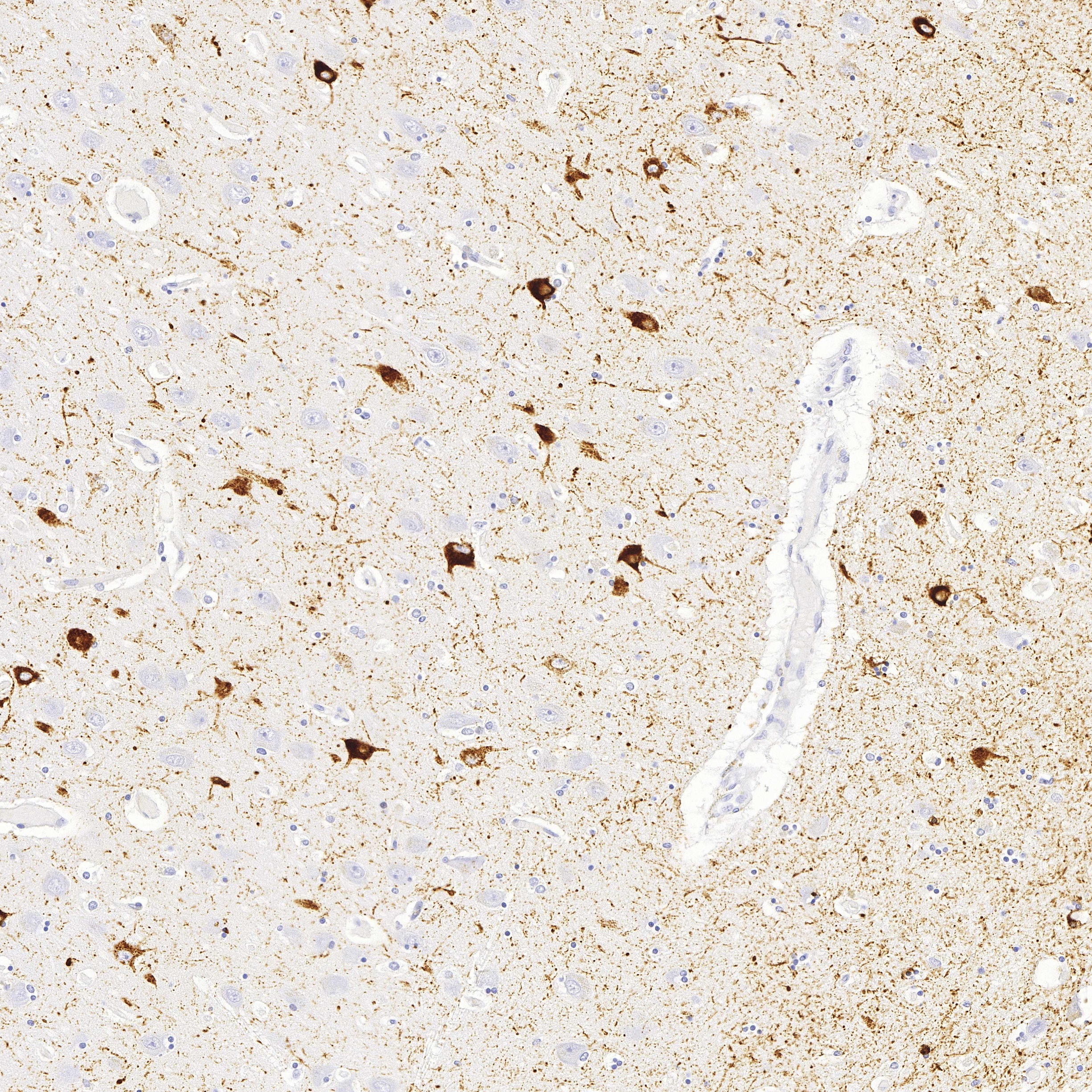

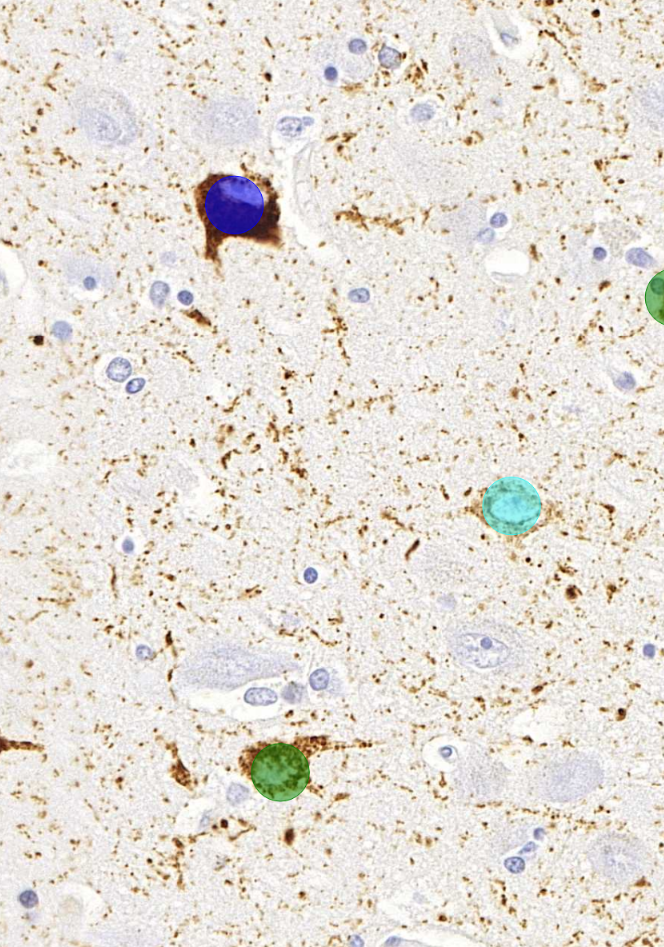


**(D)**

**(C)**

**(B)**

**(A)**

**Supplementary Figure 1.**Training of the AI model. (A) A tissue layer was first trained to identify tissue from background. Under the tissue layer, the NFT layer was trained to identify NFTs in three classes: pretangles (light blue), intermediary NFTs (green), and mature NFTs (blue). (B) Image of the AI model identifying tissue and NFTs in stratum pyramidale of the CA1 subfield in a study participant. (C-D) Example of the AI model identifying three NFTs as different maturity classes.

**1.4 Segmentation of hippocampal subfields**

The hippocampal subregions were drawn manually on the hippocampal WSIs in Aiforia Cloud by segmenting them similarly to a recent study [3]. Two medical textbooks were also used as guidance [4,5]. Corresponding hippocampal WSIs of hematoxylin and eosin-stained slides were used as guidance for segmentation. Of the dentate gyrus (DG), only the stratum granulosum was segmented. CA4 was segmented as the area enveloped by the DG. The segmentation of fields CA1-CA3 was restricted to the stratum pyramidale. The CA1-CA2 border was drawn where the dense and narrow stratum pyramidale of CA2 sharply broadens and the neurons become more scattered, and the CA2-CA3 border was drawn where the tightly packed band of ovoid neurons of CA2 loosens somewhat. The CA1-subiculum border was drawn obliquely where the stratum radiatum of CA1 dissipates. The subiculum continued from CA1, ending obliquely where the small cell islands of layer II of the presubiculum appear above the distal portion of subiculum. We segmented the entorhinal cortex (EC) as the region between the parasubiculum and the collateral sulcus in the parahippocampal gyrus. However, the segmentation of the entorhinal area in this region is uncertain, as only remnants of the caudal extension of the EC are usually present at the LGN level of the posterior hippocampus [6]. This should be considered when viewing the results.


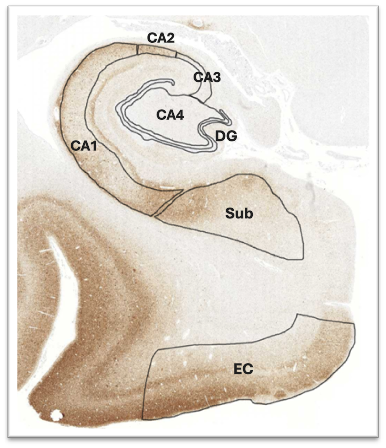


**Supplementary Figure 2.** Segmentation of hippocampal subfields on AT8-stained sections.

**2 Supplementary Results**

**2.1 Possible factors affecting disease severity in PART**

We looked at if there were any characteristics that were associated with disease severity (i.e. higher Braak stages) in the PART participants only (n=44). Supplementary Table 10 shows the cognitive status, *APOE* genotype and neuropathological features of the PART participants across Braak stages I-IV. Only 4/44 participants had Braak stage IV, and three of them were demented. None of them had Thal phase 0. There was no statistically significant correlation between Thal phases 0-2 and Braak stage severity or hippocampal NFT density in any hippocampal subfield. Cortical Aβ burden did not correlate statistically significantly with Braak stage severity either, and only NFT density in the EC showed a statistically significant correlation with cortical Aβ burden (*B* = 115.08, 95% CI 20.97 to 209.20, *P* = .018, age and sex adjusted linear regression analysis). Braak stage IV PART individuals were more likely to have CAA, LATE-NC or AGs compared with the lower Braak stage PART individuals, but the results were not statistically significant. There was also no statistically significant association between PART Braak stage severity and LRP or different types of ARTAG. However, the low number of PART cases with Braak stage IV affects the reliability of these comparisons.

**2.2 Thal 3/CERAD 0 individuals**

Current guidelines preferentially define definite PART as Thal phase 0 and possible PART as Thal phase1-2, but alternatively CERAD score 0 and 1 can be used, respectively [7]. Individuals with possible PART represent an interface where it is not known if they will evolve into AD and what threshold for Aβ should be used when defining them. In the present study, we excluded 9 Thal 3/CERAD 0 individuals from our study groups because they did not fit our grouping criteria. These individuals showed some “PART-like” features, such as a lack of APOE-ε4, less dementia, and 4/9 belonged to the 90th percentile of CA2/CA1 values (Supplementary Table 1, Supplementary Table 6). Unlike the PART individuals, most of them did however show some degree of LATE-NC (7/9) and CAA (8/9) (Supplementary Table 1). The Thal 3/CERAD 0 individuals were also noted by Walker et al as having higher CA2/CA1 ratios than Thal 3 cases with neuritic plaques, stating that “Thal phase 3 cases lie on a border where they could represent AD-related or PART pathology” [8]. Thus, these individuals pose a challenge when defining PART.

**Supplementary Table 1.** Demographics, neuropathological features, and *APOE* and dementia status separately for definite PART, possible PART, and Thal 3/CERAD 0 individuals.

|  | | Definite PART | Possible PART | Thal 3, CERAD 0 |
| --- | --- | --- | --- | --- |
| Total, n* |  | 13 | 31 | 9 |
| Sex | Male | 2 (15.4) | 7 (22.6) | 3 (33.3) |
|  | Female | 11 (84.6) | 24 (77.4) | 6 (66.7) |
| Age at death (median), years |  | 91 | 92.2 | 91 |
| Thal phase | 0 | 13 (100.0) | 0 | 0 |
|  | 1-2 | 0 | 31 (100.0) | 0 |
|  | 3 | 0 | 0 | 9 (100.0) |
|  | 4-5 | 0 | 0 | 0 |
| CERAD score | 0 | 13(100.0) | 31(100.0) | 9 (100.0) |
|  | 1 | 0 | 0 | 0 |
|  | 2 | 0 | 0 | 0 |
|  | 3 | 0 | 0 | 0 |
| Braak stage | I-II | 7 (53.8) | 10 (32.3) | 4 (44.4) |
|  | III-IV | 6 (46.2) | 21 (67.7) | 5 (55.6) |
|  | V-VI | 0 | 0 | 0 |
| LRP | none | 12 (92.3) | 18 (58.1) | 6 (66.7) |
|  | olfactory only | 0 | 0 | 0 |
|  | amygdala predominant | 0 | 0 | 1 (11.1) |
|  | nonclassifiable | 0 | 0 | 1 (11.1) |
|  | brainstem predominant | 1 (7.7) | 3 (9.7) | 1 (11.1) |
|  | limbic | 0 | 7 (22.6) | 0 |
|  | diffuse neocortical | 0 | 3 (9.7) | 0 |
| LATE-NC | 0 | 9 (69.2) | 15 (60.0) | 2 (22.2) |
|  | 1a | 1 (7.7) | 2 (8.0) | 1 (11.1) |
|  | 1b | 0 | 2(8.0) | 0 |
|  | 1c | 2 (15.4) | 2(8.0) | 1 (11.1) |
|  | 2 | 1 (7.7) | 2(8.0) | 5 (55.6) |
|  | 3 | 0 | 2(8.0) | 0 |
| CAA | None | 13 (100.0) | 20 (64.5) | 1 (11.1) |
|  | Type 1 | 0 | 1 (3.2) | 1 (11.1) |
|  | Type 2 | 0 | 10 (32.3) | 7 (77.8) |
| ARTAG (HC) | Any | 1 (7.7) | 15 (50.0) | 1 (11.1) |
|  | Subependymal TSA | 0 | 5 (16.7) | 0 |
|  | Subpial TSA | 1(7.7) | 7 (23.3) | 1 (11.1) |
|  | Perivascular TSA | 0 | 6 (20.0) | 0 |
|  | WM TSA | 0 | 4 (13.3) | 1 (11.1) |
|  | GM ARTAG | 1 (7.7) | 9 (30.0) | 0 |
|  | WM g | 0 | 5 (16.7) | 1 (11.1) |
| AGs | No | 10 (76.9) | 19 (61.3) | 7 (77.8) |
|  | Yes | 3 (23.1) | 12 (38.7) | 2 (22.2) |
| *APOE* genotypes | 22 | 1 (8.3) | 0 | 0 |
|  | 23 | 1 (8.3) | 9 (29.0) | 3 (42.9) |
|  | 33 | 10 (83.3) | 19 (61.3) | 4 (57.1) |
|  | 24 | 0 | 1 (3.2) | 0 |
|  | 34 | 0 | 2 (6.5) | 0 |
|  | 44 | 0 | 0 | 0 |
| Dementia | No | 6 (46.2) | 20 (64.5) | 7 (77.8) |
|  | Yes | 7 (53.8) | 11 (35.5) | 2 (22.2) |

Abbreviations: AGs = argyrophilic grains; *APOE* = *apolipoprotein E*; ARTAG = aging-related tau astrogliopathy ; CAA = cerebral amyloid angiopathy; CERAD = Consortium to Establish a Registry for Alzheimer’s Disease; GM = gray matter; HC = hippocampus; LATE-NC = limbic-predominant age-related TDP-43 encephalopathy neuropathologic change; LRP = Lewy-related pathology; PART = primary age-related tauopathy*,* TSA = thorn-shaped astrocytes; WM = white matter.

*Data was available for all participants except for the following variables: LATE-NC (n=47/53), ARTAG (n=52/53), and *APOE* status (n= 50/53).

All values are n (%) unless otherwise indicated.

**Supplementary Table 2.** Results of linear regression analyses for the hippocampal subfields CA1, CA2, and EC, comparing PART and ADNC groups.

|  | **CA1** | **CA2** | **EC** |
| --- | --- | --- | --- |
|  | ***B* (95% CI), *P*-value** | ***B* (95% CI), *P*-value** | ***B* (95% CI), *P*-value** |
| **Age- and sex-adjusted analysis** |  |  |  |
| PART vs. low ADNC | NS | NS | NS |
| PART vs. moderate ADNC | 10.02 (1.39 to 18.65), *P* = .023 | NS | 5.90 (2.24 to 9.56), *P* =.002 |
| PART vs. high ADNC | 32.91 (23.96 to 41.87), *P* < .001 | 25.60 (11.68 to 39.53), *P* < .001 | 16.04 (12.19 to 19.89), *P* < .001 |
| **Adjusted for age, sex, and AGs** |  |  |  |
| PART vs. low ADNC | NS | NS | NS |
| PART vs. moderate ADNC | 10.91 (2.40 to 19.42), *P* = .012 | NS | 5.98 (2.30 to 9.66), *P* = .002 |
| PART vs. high ADNC | 35.07(26.14 to 44.01), *P* < .001 | 27.81 (13.75 to 41.87), *P* < .001 | 16.30 (12.38 to 20.21) *P* < .001 |
| AGs | 10.42 (3.05 to 17.79) *P* = .006 | NS | NS |

Abbreviations: ADNC = Alzheimer’s disease neuropathologic change; AGs = argyrophilic grains; *B* = unstandardized regression coefficient; CI = confidence interval; EC = entorhinal cortex; NS = not significant; PART = primary age-related tauopathy.

Data are visualized in Figures 2A, 2C, and 2E.

Adjusting for LATE-NC and LRP made no significant difference to the age- and sex-adjusted results (not shown).

**Supplementary Table 3.** Results of linear regression analyses with NFT density in the hippocampal subfields CA1, CA2, and EC as dependent variables, comparing PART with low and moderate ADNC in Braak stage II only.

| **Braak II only** | **CA1** | **CA2** | **EC** |
| --- | --- | --- | --- |
|  | ***B* (95% CI), *P*-value** | ***B* (95% CI), *P*-value** | ***B* (95% CI), *P*-value** |
| **Age and sex adjusted analysis** |  |  |  |
| PART vs. low ADNC | NS | NS | 5.10 (0.97 to 9.22), *P* = .018 |
| PART vs. moderate ADNC | NS | -31.37 (-55.94 to -6.81), *P* = .014 | NS |
| **adjusted for age, sex, and AGs** |  |  |  |
| PART vs. low ADNC | NS | NS | 4.42 (1.29 to 7.54), *P* = 0.008 |
| PART vs. moderate ADNC | NS | -32.45 (-58.49 to -6.41), *P* = .017 | 3.02 (0.26 to 45.77), *P* = .034 |
| AGs | 15.53 (7.50 to 23.56), *P* < .001 | NS | 5.74 (2.87 to 8.62), *P* < .001 |

Abbreviations: ADNC = Alzheimer’s disease neuropathologic change; AGs = argyrophilic grains; *B* = unstandardized regression coefficient; CI = confidence interval; EC = entorhinal cortex; NS = not significant; PART = primary age-related tauopathy*.*

Data are visualized in Figures 2B and D.

Adjusting for LATE-NC and LRP made no significant difference to the age- and sex-adjusted results (not shown).

**Supplementary Table 4.** Results of unadjusted and adjusted linear regression analysis with cortical Aβ burden as independent variable and NFT densities of hippocampal subfields as dependent variable. Cases with Braak stages I-IV are included.

|  | **Cortical Aβ burden** |
| --- | --- |
|  | ***B* (95% CI), *P*-value** |
| **Unadjusted** |  |
| EC NFT/mm2 | 1.28 (0.69 to 1.88), *P* < .001 |
| CA1 NFT/mm2 | NS (*P* = .071) |
| CA2 NFT/mm2 | NS |
| CA3 NFT/mm2 | 0.89 (0.034 to 1.75), *P* = .042 |
| CA4 NFT/mm2 | 0.55 (0.12 to 0.98), *P* = .013 |
| Sub NFT/mm2 | NS (*P* = .065) |
| DG NFT/mm2 | NS |
| **Age- and sex-adjusted** |  |
| EC NFT/mm2 | 1.22 (0.62 to 1.82), *P* < .001 |
| CA1 NFT/mm2 | NS (*P* = .089) |
| CA2 NFT/mm2 | NS |
| CA3 NFT/mm2 | 0.89 (0.02 to 1.76), *P* = .044 |
| CA4 NFT/mm2 | 0.53 (0.09 to 0.96), *P* = .017 |
| Sub NFT/mm2 | NS (*P* = .092) |
| DG NFT/mm2 | NS |
| **Adjusted for age, sex, and AGs** |  |
| EC NFT/mm2 | 1.30 (0.69 to 1.90), *P* < .001 |
| CA1 NFT/mm2 | 2.10 (0.38 to 3.64), *P* =.016 |
| CA2 NFT/mm2 | NS |
| CA3 NFT/mm2 | 0.96 (0.08 to 1.84), *P* = .034 |
| CA4 NFT/mm2 | 0.53 (0.09 to 0.97), *P* = .020 |
| Sub NFT/mm2 | 1.41 (0.70 to 2.11), *P* < .001 |
| DG NFT/mm2 | NS (*P* = .062) |
| **Adjusted for age, sex, and LRP** |  |
| EC NFT/mm2 | 1.27 (0.67 to 1.88), *P* < .001 |
| CA1 NFT/mm2 | NS |
| CA2 NFT/mm2 | NS |
| CA3 NFT/mm2 | NS (*P* = .050) |
| CA4 NFT/mm2 | 0.54 (0.10 to 0.98), *P* = .017 |
| Sub NFT/mm2 | NS |
| DG NFT/mm2 | NS |
| **Adjusted for age, sex, and LATE-NC** |  |
| EC NFT/mm2 | 1.13 (0.54 to 1.73), *P* < .001 |
| CA1 NFT/mm2 | NS |
| CA2 NFT/mm2 | NS |
| CA3 NFT/mm2 | NS |
| CA4 NFT/mm2 | NS (*P* = .057) |
| Sub NFT/mm2 | NS |
| DG NFT/mm2 | NS |

Abbreviations: AGs = argyrophilic grains; *B* = unstandardized regression coefficient; CI = confidence interval; DG = dentate gyrus; EC = entorhinal cortex; LATE-NC = limbic-predominant age-related TDP-43 encephalopathy neuropathologic change; LRP = Lewy-related pathology; NFT = neurofibrillary tangles; NS = not significant; Sub = subiculum*.*

Data are visualized in Figure 3.

**Supplementary Table 5.** Results of linear regression analysis with CA2/CA1 as dependent variable, comparing PART with ADNC groups.

|  | **CA2/CA1 ratio** |
| --- | --- |
|  | ***B* (95% CI), *P*-value** |
| **Age- and sex-adjusted analysis** |  |
| PART vs. low ADNC | NS |
| PART vs. moderate ADNC | -1.16 (-2.35 to 0.016), *P* = 0.053* |
| PART vs. high ADNC | -2.04 (-3.26 to -0.81), *P* = .001 |
| **Adjusted for age, sex, and AGs** |  |
| PART vs. low ADNC | NS |
| PART vs. moderate ADNC | -1.27 (-2.44 to -1.07), *P* = .033 |
| PART vs. high ADNC | -2.30 (-3.52 to -1.07), *P* < .001 |
| AGs | -1.28 (-2.30 to -0.27) *P* = .013 |

Abbreviations: ADNC = Alzheimer’s disease neuropathologic change; AGs = argyrophilic grains; *B* = unstandardized regression coefficient; CI = confidence interval; NS = not significant; PART = primary age-related tauopathy.

*borderline significance

Data are visualized in Figure 4E.

Adjusting for LATE-NC and LRP made no significant difference to the age- and sex-adjusted results (not shown).

**Supplementary Table 6**. Features of the CA2/CA1 outliers, (i.e. the 90^th^ percentile of values).

|  | | ≥ 90th percentile | | < 90th percentile | | *P-*value*** |
| --- | --- | --- | --- | --- | --- | --- |
|  |  | n | % | n | % |  |
| Total |  | 20 | 100 | 181 | 100 |  |
| Braak stage | II | 14 | 70 | 24 | 13 | < .001 |
|  | III-IV | 6 | 30 | 95 | 53 |  |
|  | V-VI | 0 | 0 | 62 | 34 |  |
| CERAD score | 0 | 13 | 65 | 41 | 23 | < .001 |
|  | 1 | 3 | 15 | 16 | 9 |  |
|  | 2 | 4 | 20 | 104 | 57 |  |
|  | 3 | 0 | 0 | 20 | 11 |  |
| Thal phase | 0 | 5 | 25 | 8 | 4 | < .001 |
|  | 1-2 | 4 | 20 | 32 | 18 |  |
|  | 3 | 7 | 35 | 16 | 9 |  |
|  | 4-5 | 4 | 20 | 125 | 69 |  |
| AGs | No | 19 | 95 | 129 | 72 | .029 |
|  | Yes | 1 | 5 | 51 | 28 |  |
| Study group^†^ | PART | 9 | 56 | 34 | 20 | < .001 |
|  | low ADNC | 3 | 19 | 16 | 9 |  |
|  | moderate ADNC | 4 | 25 | 64 | 37 |  |
|  | high ADNC | 0 | 0 | 60 | 34 |  |
| *APOE* ε4 allele | No | 18 | 95 | 112 | 68 | .015 |
|  | Yes | 1 | 5 | 53 | 32 |  |

Abbreviations: ADNC = Alzheimer’s disease neuropathologic change; AGs = argyrophilic grains; *APOE* = *apolipoprotein E*; CERAD = Consortium to Establish a Registry for Alzheimer’s Disease; PART = primary age-related tauopathy*.*

*Fisher's exact test was used.

^†^The remaining four individuals had Thal phase 3 and CERAD score 0 and were excluded from the study groups.

**Supplementary Table 7.** Results of linear regression analysis with preNFT, iNFT, and mNFT densities of all hippocampal subfields as dependent variable, comparing CERAD score 0 vs. 1 and 0 vs. 2-3.

|  | **CERAD 0 vs. 1***  *B* (95% CI), *P*-value | **CERAD 0 vs. 2-3^†^**  *B* (95% CI), *P*-value | **CERAD 0 vs. 2-3^‡^**  *B* (95% CI), *P*-value |
| --- | --- | --- | --- |
| **EC** |  |  |  |
| preNFT/mm^2^ | NS | NS | 0.30 (0.06 to 0.53), *P* = .015 |
| iNFT/mm^2^ | NS | 3.80 (2.30 to 5.31), *P* < .001 |  |
| mNFT/mm^2^ | NS | 5.66 (3.44 to 7.88), *P* < .001 |  |
| **CA1** |  |  |  |
| preNFT/mm^2^ | NS | -1.28 (-2.51 to -0.04) *P* = .043 | NS |
| iNFT/mm^2^ | NS | 8.11 (4.68 to 11.54), *P* < .001 |  |
| mNFT/mm^2^ | NS | 15.12 (9.28 to 20.96), *P* < .001 |  |
| **CA2** |  |  |  |
| preNFT/mm^2^ | NS | NS |  |
| iNFT/mm^2^ | NS | NS |  |
| mNFT/mm^2^ | NS | 15.98 (8.02 to 23.95), *P* < .001 |  |
| **CA3** |  |  |  |
| preNFT/mm^2^ | NS | NS |  |
| iNFT/mm^2^ | NS | 2.91 (0.74 to 5.08), *P* = .009 |  |
| mNFT/mm^2^ | NS | 5.56 (2.62 to 8.51), *P* <.001 |  |
| **CA4** |  |  |  |
| preNFT/mm^2^ | NS | 0.62 (0.32 to 0.92), *P* <.001 |  |
| iNFT/mm^2^ | NS | 1.81 (0.66 to 2.96), *P* = .002 |  |
| mNFT/mm^2^ | NS | 2.86 (1.41 to 4.30), *P* < .001 |  |
| **Subiculum** |  |  |  |
| preNFT/mm^2^ | NS | -0.93 (-1.84 to -0.01), *P* = .047 | NS |
| iNFT/mm^2^ | NS | 3.88 (2.00 to 5.75), *P* <.001 |  |
| mNFT/mm^2^ | NS | 5.63 (2.87 to 8.40), *P* < .001 |  |
| **DG** |  |  |  |
| preNFT/mm^2^ | NS | 3.09 (1.32 to 4.87), *P* < .001 |  |
| iNFT/mm^2^ | NS | 7.14 (1.38 to 12.90), *P* = .015 |  |
| mNFT/mm^2^ | NS | 4.99 (1.70 to 8.29), *P* = .003 |  |

Abbreviations: AGs = argyrophilic grains; *B* = unstandardized regression coefficient; CERAD = Consortium to Establish a Registry for Alzheimer’s Disease; CI = confidence interval; DG = dentate gyrus; EC = entorhinal cortex; NS = not significant*.*

*Age- and sex-adjusted. Results did not change when adding AGD as a covariate

^†^Age- and sex-adjusted.

^‡^Adjusted for age, sex, and AGD. Results are only shown when there was a change to statistical significance or a change from statistically significant to non-significant.

Data are visualized in Figure 6.

Adjusting for LATE-NC and LRP made no significant difference to the age- and sex-adjusted results (not shown).

**Supplementary Table 8**. Results of linear regression analysis with preNFT, iNFT, and mNFT densities of all hippocampal subfields as dependent variable, comparing Thal phase 0 with Thal phase 1-2, 3 and 4-5.

|  | **Age and sex adjusted** | |  | **Adjusted for age, sex and AGs*** | | **Adjusted for age, sex and LATE-NC^†^** | | |
| --- | --- | --- | --- | --- | --- | --- | --- | --- |
|  | Thal 0 vs. 1-2 | Thal 0 vs. 3 | Thal 0 vs. 4-5 | Thal 0 vs. 1-2 | Thal 0 vs. 3 | Thal 0 vs.  1-2 | Thal 0 vs. 3 | Thal 0 vs.  4-5 |
|  | *B* (95% CI),  *P*-value | *B* (95% CI), *P*-value | *B* (95% CI),  *P*-value | *B* (95% CI),  *P*-value | *B* (95% CI),  *P*-value | *B* (95% CI),  *P*-value | *B*(95% CI),  *P*-value | *B* (95% CI), *P*-value |
| **EC** |  |  |  |  |  |  |  |  |
| preNFT/mm^2^ | 0.64 (0.12 to 1.16), *P*= .017 | 0.55 (0.01 to 1.09),  *P* = .047 | 0.66 (0.19 to 1.12), *P* = .006 | NS (*P* = .052) | NS (*P* = .067) | NS  (*P* = .056) | NS  (*P* = .057) |  |
| iNFT/mm^2^ | NS | NS | 5.52 (2.84 to 8.19), *P* < .001 |  |  |  |  |  |
| mNFT/mm^2^ | NS | NS | 8.15 (4.21 to 12.09), *P* < .001 |  |  |  |  |  |
| **CA1** |  |  |  |  |  |  |  |  |
| preNFT/mm^2^ | 2.97 (0.55 to 5.39), *P* =.016 | NS | NS |  |  | NS  (*P* = .061) |  |  |
| iNFT/mm^2^ | NS | NS | 6.79 (0.57 to 13.01), *P* =.033 |  |  |  |  | NS  (*P* = .074) |
| mNFT/mm^2^ | NS | NS | 17.54 (7.05 to 28.04), *P* = .001 |  |  |  |  |  |
| **CA2** |  |  |  |  |  |  |  |  |
| preNFT/mm^2^ | 4.76 (1.40 to 8.12), *P* = .006 | NS | NS |  |  |  |  |  |
| iNFT/mm^2^ | NS | NS | NS |  |  |  |  |  |
| mNFT/mm^2^ | NS | NS | 19.48 (5.04 to 33.92), *P* =.008 |  |  |  |  |  |
| **CA3** |  |  |  |  |  |  |  |  |
| preNFT/mm^2^ | 1.38 (0.08 to 2.68), *P* = .038 | NS | 1.27 (0.09 to 2.45), *P* = .035 | NS |  | NS  (*P* = .077) |  | NS  (*P* = .081) |
| iNFT/mm^2^ | NS | NS | NS |  |  |  |  |  |
| mNFT/mm^2^ | NS | NS | NS |  |  |  |  |  |
| **CA4** |  |  |  |  |  |  |  |  |
| preNFT/mm^2^ | NS | NS | 0.89 (0.35 to 1.43), *P* = .001 |  |  |  |  |  |
| iNFT/mm^2^ | NS | NS | NS |  |  |  |  |  |
| mNFT/mm^2^ | NS | NS | NS |  |  |  |  |  |
| **Sub** |  |  |  |  |  |  |  |  |
| preNFT/mm^2^ | 2.04 (0.25 to 3.82), *P* = .025 | NS | NS | NS (*P* = .074) |  | NS |  |  |
| iNFT/mm^2^ | NS | NS | 5.15 (1.85 to 8.45), *P* = .002 |  |  |  |  |  |
| mNFT/mm^2^ | NS | NS | 7.25 (2.37 to 12.12), *P* = .004 |  |  |  |  |  |
| **DG** |  |  |  |  |  |  |  |  |
| preNFT/mm^2^ | NS | NS | 3.89 (0.71 to 7.07), *P* = .017 |  |  |  |  | NS  (*P* = .073) |
| iNFT/mm^2^ | NS | NS | NS (P = .070) |  |  |  |  |  |
| mNFT/mm^2^ | NS | NS | 6.15 (0.24 to 12.05), *P* = .042 |  |  |  |  | NS  (*P* = .080) |

Abbreviations: AGs = argyrophilic grains; *B* = unstandardized regression coefficient; CERAD = Consortium to Establish a Registry for Alzheimer’s Disease; CI = confidence interval; DG = dentate gyrus; EC = entorhinal cortex; LATE-NC = limbic-predominant age-related TDP-43 encephalopathy neuropathologic change ; NS = not significant*.*

*Results are only shown when there was a change to statistical significance or a change from statistically significant to non-significant. There was no change in statistical significances when analyzing Thal 0 vs. 4-5.

^†^Results are only shown when there was a change to statistical significance or a change from statistically significant to non-significant. Adjusting for LRP did not significantly change the results.

Data are visualized in Supplementary Figure 4.

**Supplementary Table 9.** Results of linear regression analysis with preNFT, iNFT, and mNFT proportions of total NFTs as dependent variables, comparing PART with the ADNC groups.

|  | PART vs. low ADNC* | PART vs. moderate ADNC† | PART vs. high ADNC^†^ | PART vs. moderate ADNC^‡^ | PART vs. high ADNC^‡^ |
| --- | --- | --- | --- | --- | --- |
|  | *B* (95% CI), *P*-value | *B* (95% CI), *P*-value | *B* (95% CI), *P*-value | *B* (95% CI), *P*-value | *B* (95% CI), *P*-value |
| **EC** |  |  |  |  |  |
| preNFT | NS | -0.04 (-0.07 to -0.01), *P* = .006 | -0.09 (-0.12 to -0.06), *P* < .001 |  |  |
| iNFT | NS | NS | NS |  |  |
| mNFT | NS | NS | 0.08 (0.001 to 0.16), *P* = .047 |  | NS (AGs) |
| **CA1** |  |  |  |  |  |
| preNFT | NS | NS | -0.15 (-0.20 to -0.10), *P* < .001 |  |  |
| iNFT | NS | NS | NS |  |  |
| mNFT | NS | NS | 0.18 (0.11 to 0.25), P < .001 | 0.08 (0.001 to 0.155), P = .046 (LATE-NC) |  |
| **CA2** |  |  |  |  |  |
| preNFT | NS | NS | -0.09 (-0.17 to -0.02), *P* = .012 |  | NS (*P*=.059) (LATE-NC) |
| iNFT | NS | NS | NS |  |  |
| mNFT | NS | NS | 0.16 (0.06 to 0.26), *P* = .003 |  |  |
| **CA3** |  |  |  |  |  |
| preNFT | NS | NS | NS (*P* = .051) |  | NS (AGs, LATE-NC, LRP) |
| iNFT | NS | NS | NS |  |  |
| mNFT | NS | NS | NS |  |  |
| **CA4** |  |  |  |  |  |
| preNFT | NS | NS | NS |  |  |
| iNFT | NS | NS | NS |  |  |
| mNFT | NS | NS | NS |  |  |
| **Subiculum** |  |  |  |  |  |
| preNFT | NS | -0.08 (-0.13 to -0.02), P = .005 | -0.16 (-0.22 to -0.10), *P* < .001 |  |  |
| iNFT | NS | NS | NS |  |  |
| mNFT | NS | NS | 0.12 (0.04 to 0.21), *P* = .006 |  |  |
| **DG** |  |  |  |  |  |
| preNFT | NS | NS | 0.10 (0.023 to 0.17), *P* = .023 |  |  |
| iNFT | NS | NS | NS |  |  |
| mNFT | NS | NS | NS |  |  |

Abbreviations: ADNC = Alzheimer’s disease neuropathologic change; AGs = argyrophilic grains; *B* = unstandardized regression coefficient; CI = confidence interval; DG = dentate gyrus; EC = entorhinal cortex; LATE-NC = limbic-predominant age-related TDP-43 encephalopathy neuropathologic change; LRP = Lewy-related pathology; NS = not significant; PART = primary age-related tauopathy.

*Age- and sex-adjusted. Results remained when adding AGs, LRP and LATE-NC as a covariate.

^†^Age- and sex-adjusted.

^‡^Adjusted for age, sex, and AGs / LRP / LATE-NC. Results are only shown when there was a change to statistical significance or a change from statistically significant to non-significant. Which covariate caused the change is indicated in parenthesis.

Data are visualized in Figure 7.

**Supplementary Table 10.** Cognitive status, *APOE* genotype and neuropathological features of PART participants.

|  | | Braak stage | | | | *P^†^* |
| --- | --- | --- | --- | --- | --- | --- |
|  |  | I | II | III | IV |  |
| n* |  | 1 | 16 | 23 | 4 |  |
| Dementia | No | 0 | 11 (68.7) | 14 (60.9) | 1 (25.0) | NS |
|  | Yes | 1 (100.0) | 5 (31.3) | 9 (39.1) | 3 (75.0) |  |
| MMSE (mean) | | 14 | 19 | 17 | 15 | NS |
| *ApoE* genotype | 22 | 1 (100.0) | 0 | 0 | 0 | NS |
|  | 23 | 0 | 4 (26.7) | 4 (17.4) | 2 (50.0) |  |
|  | 33 | 0 | 10 (66.7) | 17 (73.9) | 2 (50.0) |  |
|  | 24 | 0 | 0 | 1 (4.3) | 0 |  |
|  | 34 | 0 | 1 (6.7) | 1 (4.3) | 0 |  |
|  | 44 | 0 | 0 | 0 | 0 |  |
| Thal phase | 0 | 1 (100.0) | 6 (37.5) | 6 (26.1) | 0 | NS |
|  | 1 | 0 | 7 (43.8) | 8 (34.8) | 3 (75.0) |  |
|  | 2 | 0 | 3 (18.8) | 9 (39.1) | 1 (25.0) |  |
| LRP | none | 1 (100.0) | 10 (62.5) | 17 (73.9) | 2 (50.0) | NS |
|  | olfactory only | 0 | 0 | 0 | 0 |  |
|  | amygdala predominant | 0 | 0 | 0 | 0 |  |
|  | nonclassifiable | 0 | 0 | 0 | 0 |  |
|  | brainstem predominant | 0 | 2 (12.5) | 1 (4.3) | 1 (25.0) |  |
|  | limbic | 0 | 2 (12.5) | 4 (17.4) | 1 (25.0) |  |
|  | diffuse neocortical | 0 | 2 (12.5) | 1 (4.3) | 0 |  |
| CAA | none | 1 (100.0) | 12 (75.0) | 19 (82.6) | 1 (25.0) | NS (.085) |
|  | Type 1 | 0 | 0 | 0 | 1 (25.0) |  |
|  | Type 2 | 0 | 4 (25.0) | 4 (17.4) | 2 (50.0) |  |
| AGs | No | 1 (100.0) | 12 (75.0) | 15 (65.2) | 1 (25.0) | NS |
|  | Yes | 0 | 4 (25.0) | 8 (34.8) | 3 (75.0) |  |
| ARTAG | None | 1 (100.0 | 11 (68.8) | 14 (60.9) | 1 (33.3) | NS |
|  | Any | 0 | 5 (31.3) | 9 (39.1) | 2 (66.7) |  |
|  | Subependymal TSA | 0 | 1 (6.3) | 2 (8.7) | 2 (66.7) | NS (.081) |
|  | Subpial TSA | 0 | 3 (18.8) | 4 (17.4) | 1 (33.3) | NS |
|  | Perivascular TSA | 0 | 1 (6.3) | 3 (13.0) | 2 (66.7) | NS (.074) |
|  | WM TSA | 0 | 1 (6.3) | 2 (8.7) | 1 (33.3) | NS |
|  | GM ARTAG | 0 | 5 (31.3) | 5 (21.7) | 0 | NS |
|  | WM ARTAG | 0 | 1 (6.3) | 3 (13.0) | 1 (33.3) | NS |
| LATE-NC | 0 | 1 (100.0) | 10 (62.5) | 12 (54.5) | 1 (25.0) | NS |
|  | 1a | 0 | 0 | 2 (9.1) | 1 (25.0) |  |
|  | 1b | 0 | 0 | 2 (9.1) | 0 |  |
|  | 1c | 0 | 3 (18.8) | 1 (4.5) | 0 |  |
|  | 2 | 0 | 1 (6.3) | 4 (18.2) | 2 (50.0) |  |
|  | 3 | 0 | 2 (12.5) | 1 (4.5) | 0 |  |
| Arteriolosclerosis  (SI), mean | Amygdala | 0.41 | 0.33 | 0.36 | 0.36 | NS |
|  | Hippocampus | 0.35 | 0.36 | 0.36 | 0.42 | NS |
|  | Frontal white matter | 0.54 | 0.37 | 0.38 | 0.40 | NS |

Abbreviations: AGs = argyrophilic grains; *APOE* = apolipoprotein E; ARTAG = aging-related tau astrogliopathy; CAA = cerebral amyloid angiopathy; TSA = thorn-shaped astrocytes; WM = white matter; GM= gray matter; LATE-NC = limbic-predominant age-related TDP-43 encephalopathy neuropathologic change; LRP = Lewy-related pathology; MMSE = Mini-Mental State Examination; NS, not significant; PART = primary age-related tauopathy; SI = sclerotic index; TSA = thorn-shaped astrocytes; WM = white matter

*All PART participants included in the study (n=44), i.e. participants with Braak stage I-IV, CERAD score 0 and Thal phase 0-2. Data was available for analysis for all participants except for the following variables: MMSE (n=42/44), LATE-NC (n=43/44), ARTAG (n=43/44), APOE (n=43/44), arteriolosclerosis in amygdala = 43/44, arteriolosclerosis in frontal white matter (n=38/44).

^†^Fisher’s exact test was used for categorical variables, and linear regression analysis for continuous variables.

All values are n (%) unless otherwise indicated.

**Supplementary table 11.** Results of regression analyses to determine predictors of cognitive decline in PART participants.

| **Independent variable** | **Dementia^‡^** | **MMSE^§^** |
| --- | --- | --- |
|  | **OR (95% CI), *P*** | **B (95% CI), *P*** |
| **Univariate analysis*** |  |  |
| Braak stage | NS | NS |
| CA1 NFTs/mm2^†^ | NS (P = .066) | -0.17 (-0.32 to -0.01), 0.039 |
| LRP of any type | NS | NS |
| LATE-NC stages 1a or 2 or 3 | NS | NS |
| CAA | NS | NS |
| AGs | NS | NS |
| ARTAG | NS | NS |
| Arteriolosclerosis (SI)^¶^ |  |  |
| Hippocampus | NS | NS (*P* = .097) |
| Amygdala | NS | NS |
| Frontal white matter | 2.73 (1.28 to 5.81), .009 | NS |

Abbreviations: AGs = argyrophilic grains; ARTAG = aging-related tau astrogliopathy; B = unstandardized coefficient B; CAA = cerebral amyloid angiopathy; CI = confidence interval; LATE-NC = limbic-predominant age-related TDP-43 encephalopathy neuropathologic change; LRP = Lewy-related pathology; MMSE = Mini-Mental State Examination; NFTs = neurofibrillary tangles; NS, not significant; OR = odds ratio; SI = sclerotic index.

*All analyses were adjusted for age and sex.

^†^All other hippocampal subfields showed no statistical significance.

^‡^Binary logistic regression analyses with dementia (yes/no) as the dependent variable and the variables seen in the leftmost column as independent variables.

^§^Linear regression analysis with the last MMSE before death as dependent variable and the variables seen in the leftmost column as independent variables.

^¶^Associations with dementia and the last MMSE before death were analyzed using categorical percentile group variables (quartiles) of arteriolosclerosis [9].


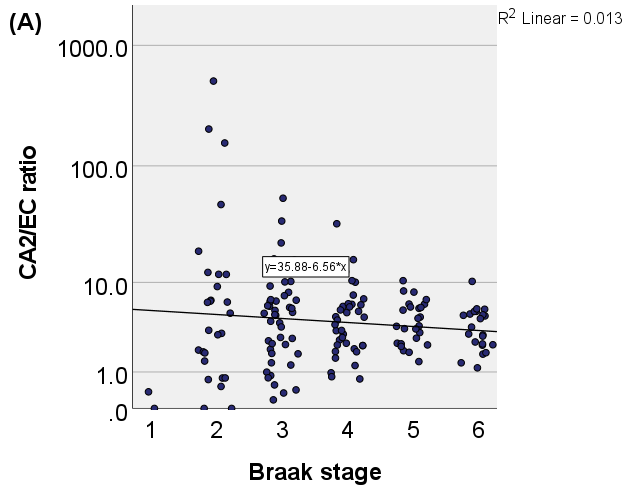

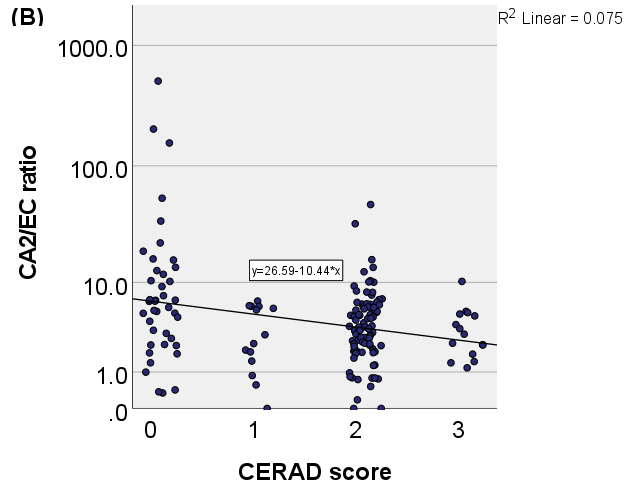


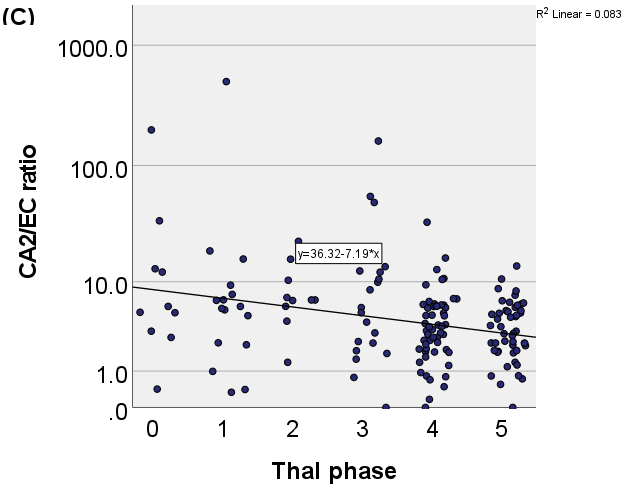

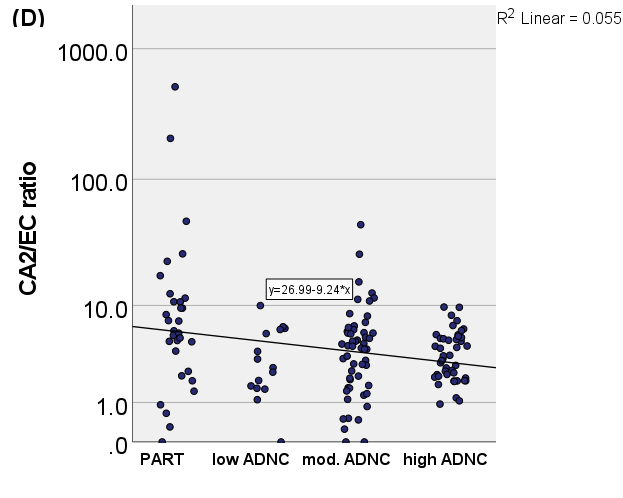


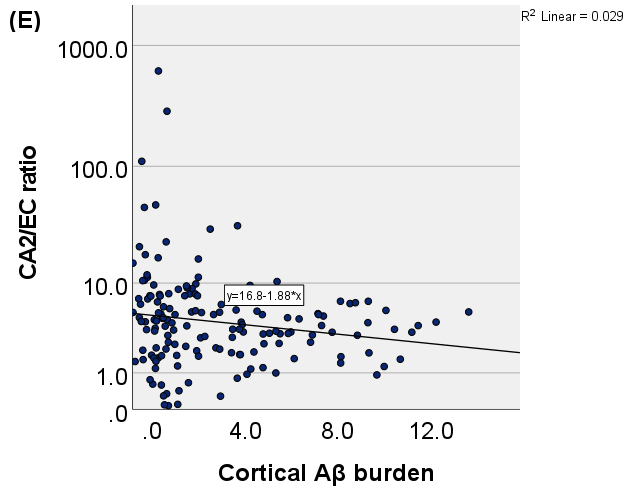


**Supplementary Figure 3.** The CA2/EC ratio showed an inverse association with (A) Braak stage (*B* = -6.08, 95% CI -12.01 to -0.16, *P* = .044), (B) CERAD score (*B* = -9.93, 95% CI -17.83 to -2.04, *P* = .014), and (C) Thal phase (*B = -*6.97, 95% CI -11.99 to -1.96, *P* = .007). (D) Moderate and high ADNC showed statistically significantly lower CA2/EC ratios compared with PART (*B* = -28.26, 95% CI -50.31 to -6.22, *P* = .012 and *B* = -27.38, 95% CI  -50.59 to -4.17, *P* = .021, respectively). (E) For cortical Aβ burden the result was not statistically significant.


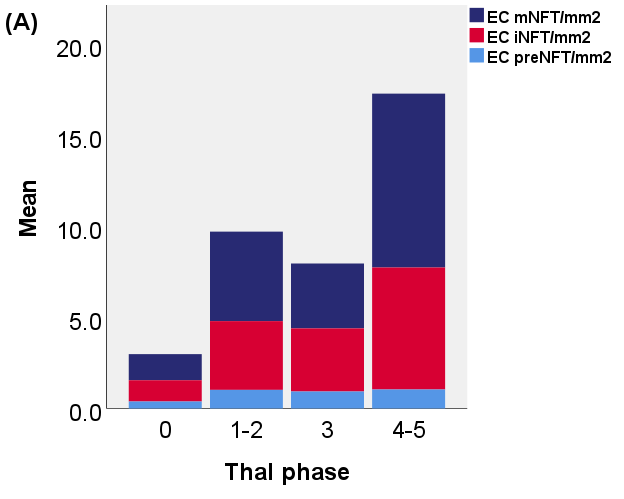

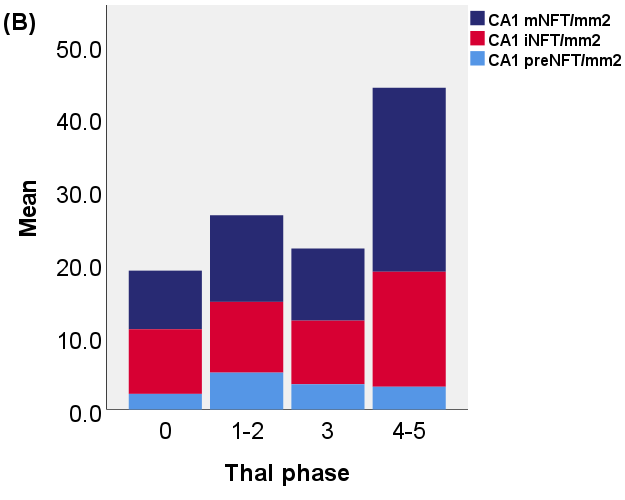


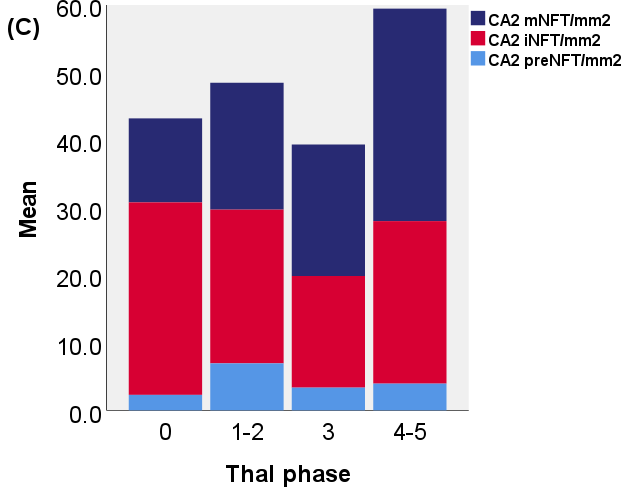

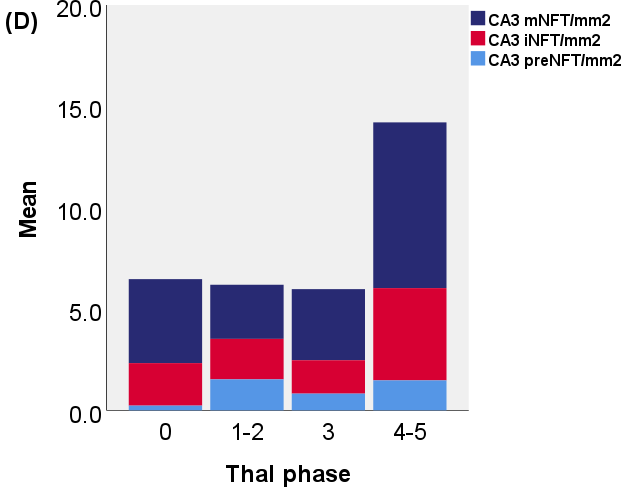


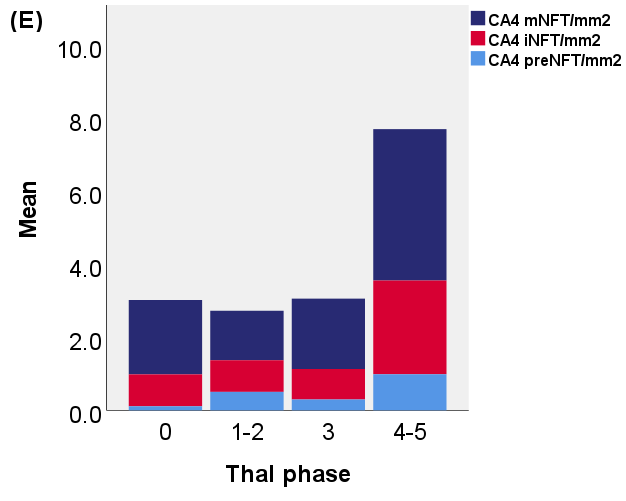

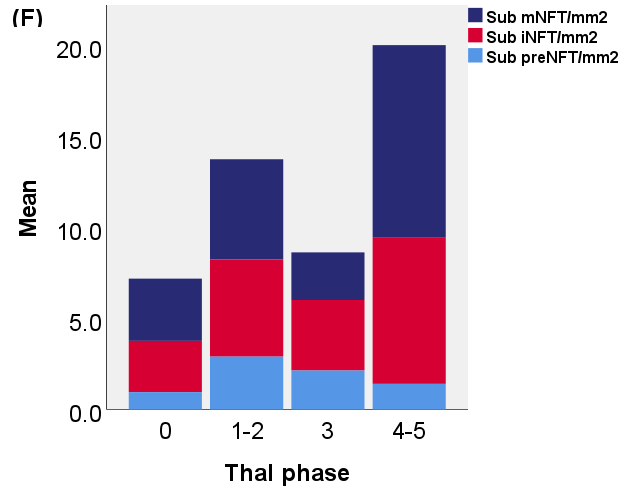

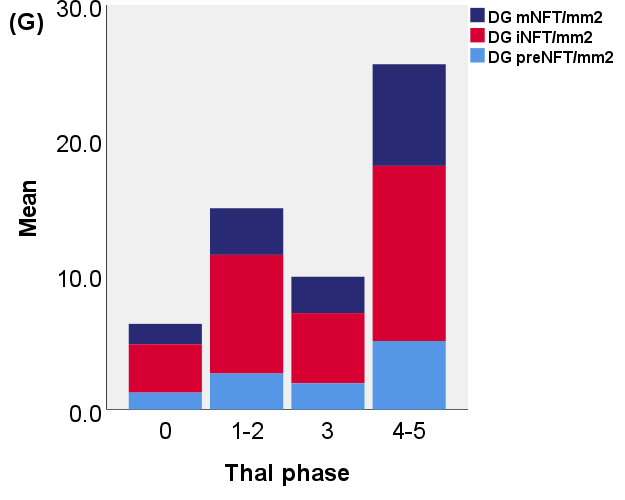


**Supplementary Figure 4.** NFT maturity levels (mean preNFT, iNFT, and mNFT density) across Thal phases in the (A) EC, (B) CA1 subfield, (C) CA2 subfield, (D) CA3 subfield, (E) CA4 subfield, (F) subiculum, and (G) DG.


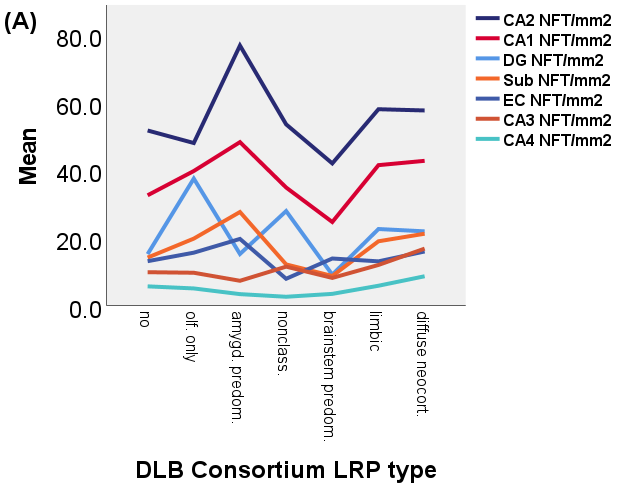

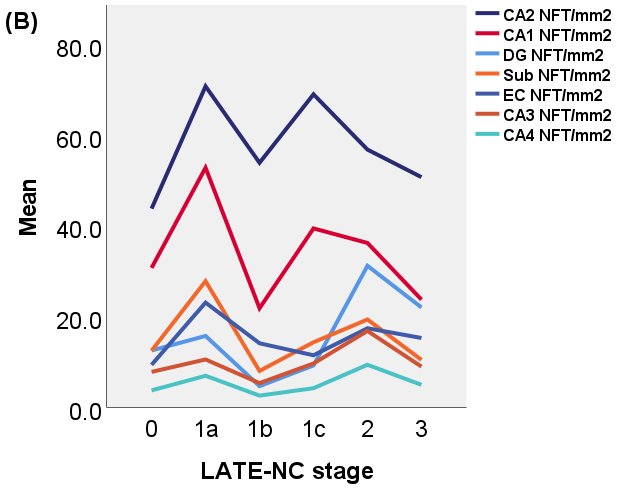


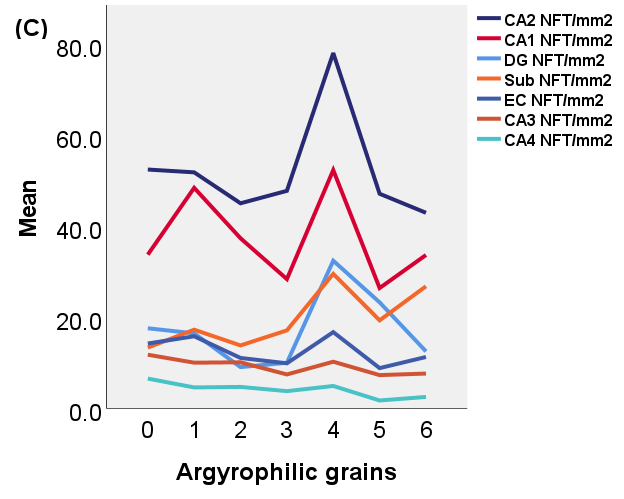


**Supplementary Figure 5.** Mean NFT density in each hippocampal subregion across (A) DLB consortium LRP type, (B) LATE-NC stage, and (C) AGs.

**References**

[1] Kovacs GG, Ferrer I, Grinberg LT, Alafuzoff I, Attems J, Budka H, et al. Aging-related tau astrogliopathy (ARTAG): harmonized evaluation strategy. Acta Neuropathol 2016;131:87–102. https://doi.org/10.1007/s00401-015-1509-x.

[2] Moloney CM, Lowe VJ, Murray ME. Visualization of neurofibrillary tangle maturity in Alzheimer’s disease: A clinicopathologic perspective for biomarker research. Alzheimer’s and Dementia 2021;17:1554–74. https://doi.org/10.1002/alz.12321.

[3] Arezoumandan S, Xie SX, Cousins KAQ, Mechanic-Hamilton DJ, Peterson CS, Huang CY, et al. Regional distribution and maturation of tau pathology among phenotypic variants of Alzheimer’s disease. Acta Neuropathol 2022;144:1103–16. https://doi.org/10.1007/s00401-022-02472-x.

[4] Duvernoy H, Cattin F, Risold P-Y. Structure, Functions, and Connections. In: Duvernoy HM, Cattin F, Risold P-Y, editors. The Human Hippocampus: Functional Anatomy, Vascularization and Serial Sections with MRI, Berlin, Heidelberg: Springer Berlin Heidelberg; 2013, p. 5–38. https://doi.org/10.1007/978-3-642-33603-4_3.

[5] Insausti R, Amaral DG. Chapter 24 - Hippocampal Formation. In: Mai JK, Paxinos G, editors. The Human Nervous System (Third Edition), San Diego: Academic Press; 2012, p. 896–942. https://doi.org/https://doi.org/10.1016/B978-0-12-374236-0.10024-0.

[6] Alafuzoff I, Arzberger T, Al-Sarraj S, Bodi I, Bogdanovic N, Braak H, et al. Staging of neurofibrillary pathology in Alzheimer’s disease: A study of the BrainNet Europe consortium. Brain Pathology 2008;18:484–96. https://doi.org/10.1111/j.1750-3639.2008.00147.x.

[7] Crary JF, Trojanowski JQ, Schneider JA, Abisambra JF, Abner EL, Alafuzoff I, et al. Primary age-related tauopathy (PART): a common pathology associated with human aging. Acta Neuropathol 2014;128:755–66. https://doi.org/10.1007/s00401-014-1349-0.

[8] Walker JM, Richardson TE, Farrell K, Iida MA, Foong C, Shang P, et al. Early Selective Vulnerability of the CA2 Hippocampal Subfield in Primary Age-Related Tauopathy. J Neuropathol Exp Neurol 2021;80:102–11. https://doi.org/10.1093/jnen/nlaa153.

[9] Mikhailenko E, Colangelo K, Tuimala J, Kero M, Savola S, Raunio A, et al. Limbic-predominant age-related TDP-43 encephalopathy in the oldest old: a population-based study. Brain 2024. https://doi.org/10.1093/brain/awae212.
